# Unraveling the Clinical Landscape of RNA Modification Regulators with Multi-Omics Insights in Pan-Cancer

**DOI:** 10.3390/cancers17162695

**Published:** 2025-08-19

**Authors:** Qingman Li, Jingjing Zhang, Zuyi Cao, Jiale Wang, Jiaxing Song, Xianfu Yi

**Affiliations:** 1 Department of Bioinformatics, School of Basic Medical Sciences, Tianjin Medical University, Tianjin 300070, China; tangqianqian_man@163.com (Q.L.); zjjing_1108@163.com (J.Z.); 2 The Second Hospital of Tianjin Medical University, Tianjin 300211, China; zuyi_cao@163.com; 3 Department of Immunology, School of Basic Medical Sciences, Tianjin Medical University, Tianjin 300070, China; jiale.wang@tmu.edu.cn; 4 College of Stomatology, Tianjin Medical University, Tianjin 300070, China; zifn_jiaxing@163.com

**Keywords:** RNA modifications, pan-cancer analysis, prognostic model, tumor microenvironment, precision oncology

## Abstract

**Background/Objectives:** Cancer remains a major global health challenge, with RNA modifications increasingly recognized as key regulators of tumor progression. However, integrated pan-cancer analyses across multiple modification types are limited. **Methods:** We performed a comprehensive analysis of 170 RNA modification-related genes across 33 cancer types, uncovering diverse expression, mutation, and epigenetic patterns. **Results:** Key regulators such as *IGF2BP3*, *CFI*, and *ELF3* showed cancer-specific prognostic significance. We developed an RNA Modification Score (RMS) with strong prognostic performance (AUC up to 0.92), correlating with the tumor stage, immune infiltration, and immunotherapy response. High-risk groups exhibited immune checkpoint dysregulation and enriched M1 macrophages in glioblastoma. Drug screening highlighted oncrasin-72 as a potential therapy. Validation via single-cell/spatial transcriptomics and immunohistochemistry confirmed the spatial localization of critical genes like *CFI* and *ELF3*. **Conclusions:** Our study reveals the multifaceted role of RNA modifications in cancer, providing a translational framework for personalized prognosis and therapy in precision oncology.

## Simple Summary

Cancer remains a significant global health issue, with RNA modifications playing vital roles in tumor development. This study analyzed 170 RNA modification-related genes across 33 cancer types, uncovering distinct expression and mutation patterns. The key regulators, including *IGF2BP3*, *CFI*, and *ELF3*, demonstrated notable prognostic value. A newly developed RNA Modification Score (RMS) achieved an AUC of up to 0.92, correlating with the tumor stage, immune response, and treatment outcomes. High-risk groups exhibited immune checkpoint dysregulation and increased M1 macrophages in glioblastoma. Drug screening identified oncrasin-72 as a promising therapeutic option. Validation confirmed the spatial localization of key genes, emphasizing the multifaceted roles of RNA modifications in cancer and offering potential for personalized prognostic and treatment strategies in precision oncology.

## 1. Introduction

Cancer presents a significant threat to human health due to its high incidence and mortality rates [1]. Globally, approximately 20 million new cancer cases and 9.7 million cancer deaths were recorded in 2022 [2]. The global cancer burden will continue to rise, with an estimated 35 million new cases annually by 2050—a 77% increase from 2022 [1]. While cancer types vary across populations, their occurrence and progression are often governed by shared molecular mechanisms [3]. Research indicates that tumors from different organs can exhibit overlapping characteristics, such as common oncogene activation pathways, elevated mutation rates in tumor suppressor genes, and chromosomal instability [3]. However, even tumors within the same organ can demonstrate considerable differences in their cell morphology, size, and gene expression profiles [4]. This combination of commonality and heterogeneity complicates tumorigenesis and progression, resulting in diverse mechanisms and outcomes. Despite advances in understanding the underlying regularities, spatiotemporal dynamics, and molecular complexities of tumors [5], a precise analysis of tumor commonality and heterogeneity remains a significant challenge in clinical diagnosis and treatment. These challenges are fundamental to precision medicine implementation, impacting diagnosis, treatment selection, efficacy evaluation, disease monitoring, drug resistance, and prognosis [6]. Thus, a thorough analysis of tumor commonality and heterogeneity is crucial for elucidating the dynamic evolution of tumors, from morphological heterogeneity to intricate molecular mechanisms, providing a vital theoretical basis for advancing precision medicine [7].

Pan-cancer analysis systematically examines the molecular aberrations across multiple cancer types to identify key dysregulated biological processes in cancer cells from varied lineages, revealing commonalities and specificities [8]. With the ongoing accumulation of high-throughput data from diverse tumors, pan-cancer analysis is increasingly vital for elucidating the molecular mechanisms underlying cancer development [9]. For instance, existing pan-cancer studies have constructed complex mutation networks that include subnetworks characterized by rare mutations found in nearly all cancer types, as well as genes with enriched mutations in specific types within these subnetworks [10,11,12]. This enhances our understanding of both the shared and distinct features among different cancers. Such studies provide critical scientific evidence for the exploration of novel diagnostic strategies and therapeutic approaches across various cancer types. Moreover, pan-cancer analysis can further dissect the genomic and cellular similarities and differences among cancer types, opening up new avenues for developing tumor prognostic models, optimizing clinical treatment strategies, and implementing personalized therapies [13].

Research indicates that cancer development is closely linked to not only gene mutations or deletions but also epigenetic regulation [14]. Epigenetics studies heritable and stable changes in gene expression that do not involve alterations in the nucleotide sequence. In some cases, these epigenetic changes can be a primary driving force behind carcinogenesis [15]. Recent studies have highlighted the role of the epitranscriptome as a third layer of gene regulation in modulating hallmark biological events associated with cancer [16]. Among these, RNA modifications—an essential aspect of epitranscriptomics—have gained significant attention [17]. Increasing evidence demonstrates that RNA modification mechanisms markedly change across various human cancers, underscoring their potential as pharmacological targets and diagnostic biomarkers [14]. RNA modifications regulate gene expression by influencing the structure, stability, splicing, localization, and translatability of RNA molecules [18]. As of 2024, over 170 types of RNA modifications have been identified, with common examples including m6A, m5C, and m7G [19,20]. RNA modification regulators fall into three categories: “writers” (which add modifications), “erasers” (which remove modifications), and “readers” (which recognize and bind modified nucleotides). Notably, some modifiers act on multiple types; for instance, ALKBH1 serves as an eraser of both m5C and m1A modifications. This multifunctionality likely plays a critical role in cancer development and progression by influencing the dynamic regulation of RNA modifications [21]. Research into RNA modifications and their regulatory factors has deepened recently, revealing their essential roles in tumor initiation, metastasis, metabolic regulation, the tumor immune microenvironment (TIME), and resistance to anticancer therapies [20]. Emerging evidence suggests that RNA modifications act as critical regulators of tumor–immune crosstalk by influencing both innate and adaptive immune responses. For instance, the m6A writer METTL3 can suppress cytotoxic T-cell infiltration in colorectal cancer by destabilizing the CXCL1 and IL-8 transcripts [22], whereas the eraser ALKBH5 promotes PD-L1 degradation in melanoma through YTHDF2-mediated transcript destabilization [23]. Numerous studies show that specific RNA modification factors (such as METTL3, IGF2BP3, and ALKBH5) exhibit abnormal expression in various cancers. By regulating the post-transcriptional modifications of key oncogenes or tumor suppressor genes, they influence tumor progression [14,24,25,26]. Furthermore, the aberrant regulation of these RNA modification factors affects cancer biology and offers new targets and strategies for anticancer drug development and precision medicine [14].

Numerous types of RNA modifications exist, and their distribution patterns in cancers vary significantly [18]. While substantial progress has been made in studies focusing on individual RNA modification types, these investigations are insufficient to fully elucidate the overall role of RNA modifications in cancer and their interrelationships [18]. The RNA modification patterns across different cancer types exhibit certain commonalities. For instance, IGF2BP3, an m6A reader, is highly expressed in both LUSC and nasopharyngeal carcinoma (NPC), with elevated expression correlating positively with poor prognosis [27,28]. However, RNA modifications also demonstrate significant heterogeneity among different cancers. For example, METTL3, an m6A writer, is downregulated in papillary thyroid carcinoma (PTC), where its reduction is associated with malignant progression and poor prognosis [29]. Conversely, in colorectal cancer (CRC), inhibition of METTL3 has shown significant antitumor effects [22]. This variability suggests that the function of RNA modifications may depend on the specific cancer type and tissue microenvironment. By systematically analyzing the RNA modifications across cancer types, we can uncover shared molecular mechanisms, identify pan-cancer biomarkers, and advance precision medicine, while deepening our understanding of molecular heterogeneity to inform targeted therapies. In summary, pan-cancer research on RNA modifications is poised to pave the way for innovative approaches to future pan-cancer therapies [30].

Despite advancements in pan-cancer research on RNA modifications, significant limitations persist. Most studies have primarily focused on m6A modifications, with systematic pan-cancer analyses of other RNA modification types, such as m5C, m1A, and m7G, remaining relatively scarce (Figure S1A; Table S1). As a result, the dynamic changes in the RNA modifications, their interactions, and their effects on the TIME and the therapeutic responses across different cancer types have not been fully elucidated. Therefore, there is an urgent need to enhance pan-cancer research on multiple RNA modification types. This project aims to construct a comprehensive pan-cancer model encompassing 33 cancer types, 15 RNA modifications, and 170 RNA modification regulatory factors. By systematically characterizing the clinical outcomes, immune landscapes, and drug responses across cancers, the project seeks to identify new targets and develop more effective treatment strategies for precision oncology. This will provide a more complete understanding of the roles and potential mechanisms of RNA modifications in cancer biology.

## 2. Materials and Methods

### 2.1. Pan-Cancer Multimodal Data and RNA Modification Genes

Pan-cancer multimodal data were obtained from the TCGA TARGET GTEx cohort via UCSC Xena (https://xenabrowser.net/ (accessed on 22 November 2024)). The data include raw count data and normalized gene expression data (transcripts per kilobase of exon model per million mapped reads, TPM), copy number variation (CNV) data estimated using GISTIC2, somatic mutation data, DNA methylation data (Methylation 27K and 450K), and clinical information.

External validation data were obtained from the Gene Expression Omnibus (GEO, https://www.ncbi.nlm.nih.gov/geo/ (accessed on 6 August 2025)) under the following accession numbers: adrenocortical carcinoma (ACC, GSE19750), bladder urothelial carcinoma (BLCA, GSE69795), glioblastoma multiforme (GBM, GSE83300), lung adenocarcinoma (LUAD, GSE29016), skin cutaneous melanoma (SKCM, GSE19234), and kidney renal clear cell carcinoma (KIRC, GSE22541).

RNA modification gene data were integrated from 14 recent publications (Table S2) and included 170 RNA modification-related genes (152 after deduplication) associated with 15 types of RNA modifications.

### 2.2. Analysis of Single Nucleotide Variations (SNVs)

Based on the pan-cancer somatic mutation data, non-silent mutation types for the 170 RNA modification genes were selected. For each cancer type, the mutation frequency of the RNA modification genes (calculated as the number of non-silent mutations per cancer sample count × 100) and the tumor mutation burden (TMB) were determined. The TMB was computed using the R package “maftools” (v2.18.0) and is expressed as the average number of mutations per 1 Mb of the protein-coding region in the genome.

### 2.3. Copy Number Variation Analysis

Pan-cancer CNV data were estimated using GISTIC2. For the 170 RNA modification genes, the proportions of different CNV types (amplification, deletion, normal) were calculated for each cancer type.

### 2.4. Methylation Analysis

Using whole-genome methylation data, we calculated the standard deviation (SD) of the methylation levels for the RNA modification genes across samples for each cancer type. Genes with high methylation heterogeneity (beta-value > 0.5 in specific or most cancers) were identified.

### 2.5. Differential Expression Analysis

Differential expression analysis was performed using the R package “DESeq2” (v1.42.1) on the gene expression count data for 29 cancer types, excluding LAML, MESO, DLBC, and UVM, which contained only cancer samples without the corresponding normal tissues. Candidate differentially expressed genes (DEGs) for the subsequent analyses were selected based on the criteria of FDR < 0.05 and |log2FC| > 0.5.

### 2.6. LASSO Regression for Prognostic Key Gene Screening and RMS Construction

The LASSO (least absolute shrinkage and selection operator) regression method was employed to eliminate variables lacking predictive value. Based on the candidate DEGs, the prognostic contribution of each gene was assessed using the OS data. Using the R package “glmnet” (v4.1-8) with 10-fold cross-validation, the optimal λ_min (based on the mean squared error, MSE) was selected. The coefficient profile of the feature variables was then examined to determine the gene coefficients at λ = λ_min. Ten cancer types that lacked key genes were excluded, leaving 19 cancer types for further analysis. The RNA Modification Score (RMS) was calculated using genes with nonzero coefficients as follows:

$$RMS=\sum_{i=1}^{i}\left({Exp}_{i}\times \beta_{i}\right)$$

where ${Exp}_{i}$ is the expression level of the i-th gene, and $\beta_{i}$ is the corresponding LASSO regression coefficient.

### 2.7. ROC Curve Evaluation of RMS Performance

ROC curve analysis was conducted to evaluate the accuracy of the RMS in predicting the patient survival status. The patient survival status was dichotomized using the OS as the endpoint and used as the response variable. The R package “pROC” (v1.18.5) was employed to fit the ROC curve, and the area under the curve (AUC) was calculated as an indicator of the sensitivity and specificity.

### 2.8. Survival Analysis for RMS Validation

For the 19 cancer types, samples were categorized into risk RMS groups based on the median RMS value. Kaplan–Meier survival curves were plotted, and the log-rank test was employed to assess the survival differences between the groups. Hazard ratios (HRs) and their 95% confidence intervals were calculated (with *p* < 0.05 and HR ≠ 1). Data processing and visualization were conducted using the R packages “survival” (v3.8.3) and “survminer” (v0.5.0).

### 2.9. Association Analysis Between TNM Staging and RMS Risk

TNM staging and clinical stage (Stage) data were extracted. To ensure analytical consistency, the staging subtypes were standardized: T1a and T1b were grouped into T1; Stage IIIA and Stage IIIB into Stage III. Chi-square tests were used to assess the associations of the TNM parameters (T1–T4, N0–N3, M0–M1) and Stages (I–IV) with the risk RMS groups.

### 2.10. Differential Analysis Based on Risk RMS Groups

Samples from the 19 cancer types were stratified into risk RMS groups based on the median RMS values, and differential gene expression analysis between these risk RMS groups was performed using DESeq2 (v1.42.1). Genes with significant differential expression (FDR < 0.05 and |log2FC| > 1) were selected for the subsequent functional annotation and pathway enrichment analyses.

### 2.11. Gene Set Enrichment Analysis

Based on the identified significant DEGs, both over-representation analysis (ORA) and gene set enrichment analysis (GSEA) were performed to explore the enrichment of the KEGG metabolic pathways. The R package “clusterProfiler” (v4.10.1) was utilized to calculate the adjusted *p*-value and normalized enrichment score (NES). Pathways with an adj. *p* < 0.05 were deemed significant and selected for visualization.

### 2.12. Analysis of TIME and Immunotherapy Response

To quantitatively assess the immune cell infiltration in the TIME, the R package “xCell” (v1.1.0) was first applied to the TPM of 19 cancers to calculate the immune cell enrichment scores. Subsequently, the R package “quantTIseq” (v1.14.0) was utilized to quantify the relative proportions of 10 major immune cell types, including B cells, M1/M2 macrophages, monocytes, neutrophils, natural killer cells, CD4+ and CD8+ T-cells, regulatory T-cells, and dendritic cells. Differences between the risk RMS groups were compared. To elucidate the role of M1 macrophages in the subsequent analyses, differential expression analysis was conducted between the RMS high-risk and low-risk groups. Subsequently, KEGG pathway over-representation analysis and GSEA were performed to systematically investigate the enrichment of the biological pathways. M1-associated pathways demonstrating significant correlations with cancer (*p* < 0.05) were identified and their results visualized accordingly.

To enhance the results’ reliability, immune infiltration data from multiple algorithms provided by the TIMER database (http://timer.cistrome.org/ (accessed on 25 November 2024))—including TIMER, CIBERSORT, quantTIseq, xCell, and MCPCOUNTER—were integrated. The Wilcoxon rank-sum test was employed to assess the expression differences among 51 finely classified immune cell subpopulations between the risk RMS groups. Additionally, single-sample gene set enrichment analysis (ssGSEA) was performed using the GSVA package (v1.50.5) on 28 immune cell marker gene sets (http://cis.hku.hk/TISIDB/data/download/CellReports.txt (accessed on 25 November 2024)) to quantify the immune cell infiltration for each sample [31].

For 32 immune checkpoint molecules [32,33], the Wilcoxon rank-sum test was employed to assess the expression differences between the risk RMS groups across 19 cancers. Additionally, by integrating cancer immune pathway data from GSCA (https://guolab.wchscu.cn/GSCA/#/ (accessed on 25 November 2024)), the standardized pathway scores (PASs) were calculated, and Student’s t-test compared the pathway activity differences between the risk RMS groups. To evaluate the predictive ability of the RMS in terms of the immunotherapy response, the tumor immune dysfunction and exclusion (TIDE) database quantified patients’ immune escape features, and the score distributions were compared between the risk RMS groups.

### 2.13. Prediction of Drug Sensitivity

Gene expression data from 1000 human cancer cell lines were obtained from the Genomics of Drug Sensitivity in Cancer (GDSC) database (https://www.cancerrxgene.org/ (accessed on 28 November 2024)). The R package “oncoPredict” (v1.2) was used to predict the IC50 values for 285 small-molecule drugs for each sample using machine learning algorithms (linear regression and ridge regression). The correlation coefficients between the predicted IC50 values and the RMS were calculated, with the cancer–drug combinations exhibiting high correlation coefficients (>0.6) being selected. Additionally, data from GSCALite (https://guolab.wchscu.cn/GSCA/#/ (accessed on 25 November 2024)) and GDSC were integrated to identify the top 30 drugs potentially associated with RMS-related genes.

### 2.14. Integration of Single-Cell Transcriptome Data

A single-cell transcriptome dataset of human GBM from the GEO database (GSE84465) was utilized. Using the Seurat (v5.0) pipeline, dimensionality reduction was performed for the top 30 principal components, followed by clustering analysis to delineate the cellular subpopulations. Cells were classified into seven types—astrocytes, myeloid cells, neoplastic cells, neurons, oligodendrocyte precursor cells (OPCs), oligodendrocytes, and vascular cells—adhering to the reference criteria [34].

### 2.15. Spatial Transcriptomics: Spatial Partitioning and Expression Validation

Spatial transcriptomics analysis was performed on a surgical specimen from a treatment-naïve GBM patient (no prior radiotherapy or chemotherapy) using established methods [35]. The spatial transcriptomics RNA sequencing data have been deposited in the Gene Expression Omnibus (GEO) under accession number GSE253080 (https://www.ncbi.nlm.nih.gov/geo/ (accessed on 6 August 2025)). Using Seurat (v5.1.0), we processed the data and integrated pathological partitioning—identifying five distinct regions: GBM cells, peritumoral areas, blood vessels, hemorrhage, and necrosis—to interpret the spatial gene expression patterns.

### 2.16. Immunohistochemistry and Quantitative Analysis

Immunohistochemistry slides were obtained from the Human Protein Atlas (www.proteinatlas.org (accessed on 14 January 2025)). Using ImageJ (v1.54p), we separated the DAB staining channel with the Color Deconvolution plugin and set the gray threshold values in Threshold (ELF3: 0–210; IGF2BP3 and CFI: 0–190) to identify positively stained regions. The percentage of positive area (%Area) and average optical density (AOD) were calculated, and their product was used to determine the immunoreactivity score [36]. Group differences were assessed using a one-tailed *t*-test.

## 3. Results

### 3.1. Pan-Cancer Expression and Genomic Profiles of RNA Modification Genes

We systematically collected and integrated 170 RNA modification regulatory genes linked to 15 types of RNA modifications through literature mining and database integration (see Section 2; Figure S1B,C; Tables S2 and S3). Using these data, we performed a comprehensive pan-cancer analysis across 33 common cancers (Figure S1D).

**Figure 1:**
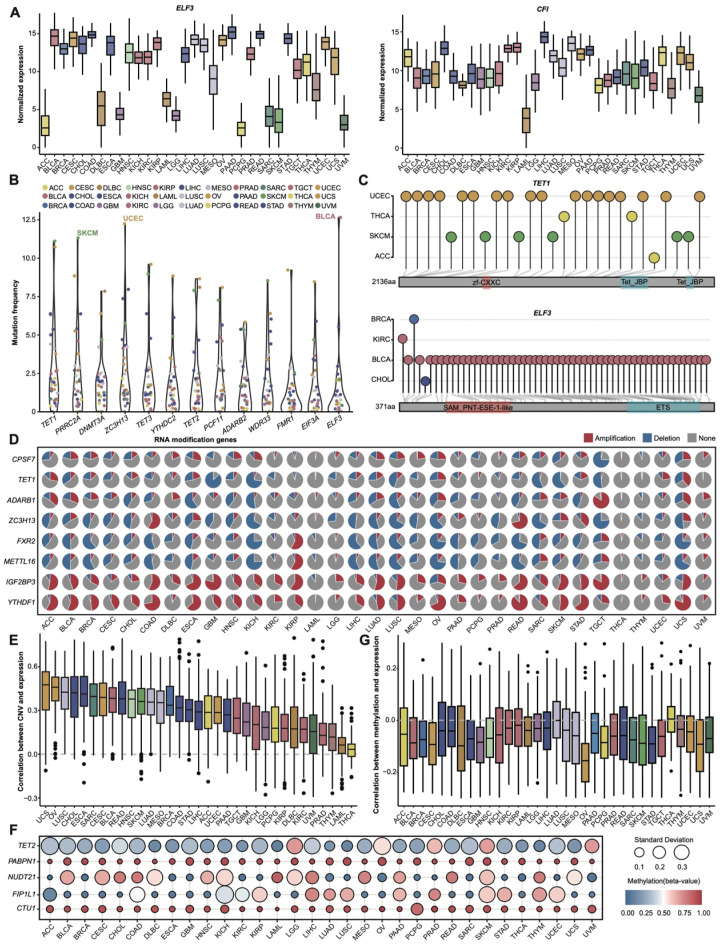
Expression and genomic landscape of RNA modification genes across 33 cancer types. (**A**) Normalized expression levels (TPM) of *ELF3* and *CFI* across 33 cancers. (**B**) Mutation frequencies of RNA modification genes (mutation frequency × 100). The top 13 genes are displayed. (**C**) Mutation site distribution of *TET1* and *ELF3* across cancers. (**D**) Copy number variation (CNV) profiles of selected RNA modification genes. (**E**) Spearman correlation analysis between CNV and RNA modification gene expression (median coefficient > 0). (**F**) Methylation levels (beta-value) and standard deviation (SD) for *TET2*, *NUDT21*, *PABPN1*, *FIP1L1*, and *CTU1*. (**G**) Correlation between methylation and RNA modification gene expression (Spearman r < 0).

RNA modification genes exhibit significant heterogeneity in their expression patterns across cancers (Figure 1A and Figure S2A,B). A few genes, such as *TRUB2* and *HNRNPA2B1*, are consistently expressed across most cancer types, primarily due to their involvement in fundamental biological processes or their roles in large biomolecular complexes. In contrast, the expression of many RNA modification genes differs markedly between cancers. For instance, *ELF3* is less expressed in ACC and UVM, while *CFI* demonstrates significantly reduced expression in LAML (Figure 1A and Figure S2A). These differential expression patterns indicate that RNA modification regulation may be specific to cancer types, highlighting the need for pan-cancer studies of the RNA modification gene expression profiles to identify potential broad-spectrum and cancer-specific regulatory targets.

We examined the mutation frequency of the RNA modification genes across various cancers. The results indicate significant variability in the mutation frequency among the 33 cancer types (Figure 1B and Figure S3A; Table S4). UCEC and SKCM demonstrate relatively high mutation burdens, with 149 and 96 mutated RNA modification genes, respectively, ranking among the top three cancers for mutation frequency. *TET1*, associated with multiple cancers [37], has the highest average mutation frequency of all the RNA modification genes, with a median of 1.768%. Notably, *ELF3*, which plays a critical role in epithelial cell differentiation and tumorigenesis, exhibits a mutation frequency of 12.65% in BLCA, significantly higher than in other cancers (Figure 1B). Comparative analyses of the mutation site distribution for *TET1* and *ELF3* reveal that *TET1* mutations are enriched in UCEC and SKCM, while *ELF3* mutation sites are significantly concentrated in BLCA (Figure 1C). These findings indicate that RNA modification genes display both common and cancer-specific patterns in their mutation frequency and site distribution.

Then, we analyzed their CNV profiles (Figure 1D; Table S5). The results indicate that certain RNA modification genes exhibit significant copy number losses in specific cancers. For example, *CPSF7* has a deletion rate of 74.67% in TGCT, while *TET1* shows 87.35% in GBM, 72.73% in KICH, and 60.49% in SKCM. Conversely, some genes display notable copy number amplifications: *ADARB1* has an amplification rate of 84.67% in TGCT, while *ZC3H13* exhibits 58.44% in COAD and 70.91% in READ (Table S3). These findings align with previous reports [38,39]. Furthermore, some genes (e.g., *FXR2*, *METTL16*) generally show copy number losses in pan-cancer, while some (e.g., *IGF2BP3*, *YTHDF1*) primarily exhibit copy number amplifications. Correlation analysis reveals a significant positive relationship between the expression levels of RNA modification genes and their CNV alterations (median correlation coefficient ≈ 0.29; Figure 1E and Figure S3B), further supporting the idea that CNVs are a crucial mechanism regulating gene expression [40].

We further analyzed the methylation patterns of associated genes. While most genes exhibit low methylation (98% with β-values < 0.5; Figure S4), distinct hypermethylation events were observed: *PABPN1* and *CTU1* show pan-cancer hypermethylation, whereas *TET2* display cancer-specific hypermethylation in PRAD (Figure 1F), aligning with prior evidence implicating *TET2* methylation as a potential biomarker for prostate cancer progression [41]. Strikingly, the methylation levels demonstrate a genome-wide negative correlation with the gene expression in 31/33 cancers (Figure 1G), reinforcing DNA methylation’s established role in transcriptional silencing.

### 3.2. RNA Modification Risk Score Model: Clinical and Biological Associations

**Figure 2:**
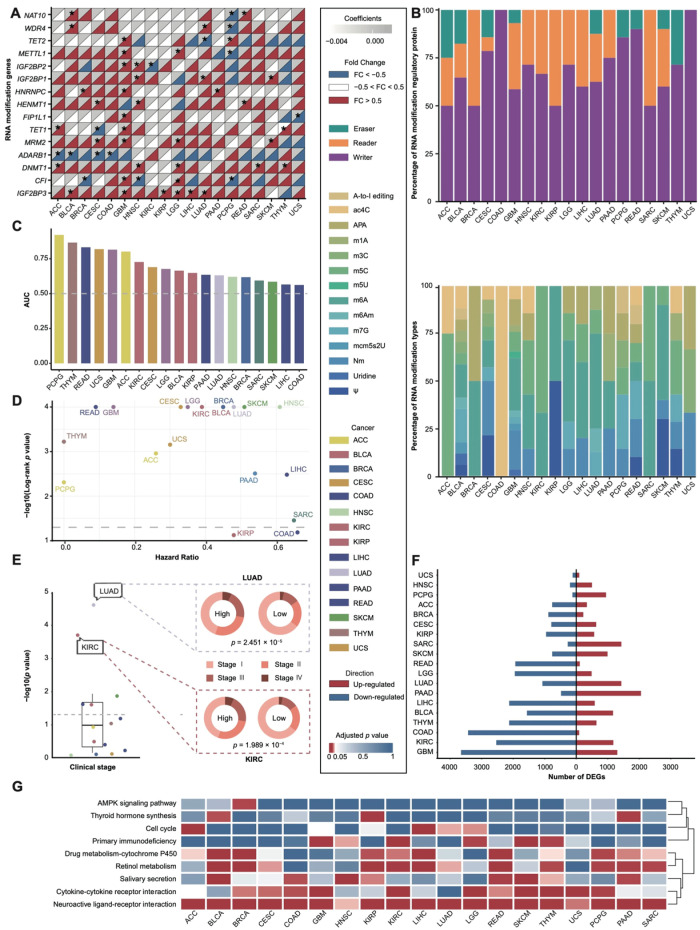
RMS construction and its clinical relevance. (**A**) Integration of differential expression analysis and LASSO regression for key gene screening. * indicates coefficients with absolute values > 0. (**B**) Statistical display of key RNA modification regulatory protein (upper) and RNA modification types (lower). (**C**) Area under the ROC curve (AUC) of RMS. Dashed line indicates AUC = 0.50. (**D**) Survival analysis validation of RMS (univariate Cox regression and Kaplan–Meier survival analysis). Dashed line indicates log-rank *p* value < 0.05. (**E**) Chi-square test for correlation between clinical stage and risk RMS groups. (**F**) Differential expression analysis based on risk RMS groups. (**G**) KEGG pathway ORA analysis between risk RMS groups in 19 cancers.

We used DESeq2 and LASSO regression to screen key genes (see Section 2). Due to the absence of paired normal tissue samples for some cancer types and the inability to identify significantly prognostic key genes in others via LASSO regression, we ultimately identified a set of prognostically relevant RNA modification genes in 19 cancers (Table S6). The results indicate that only a few RNA modification genes are relevant across multiple cancers; for example, *IGF2BP3* demonstrates significant prognostic value in six cancers (Figure 2A; Table S6) and has been suggested as a diagnostic and prognostic marker for various cancers, particularly gliomas [42]. Notably, approximately 24.6% of the identified prognostic RNA modification genes are associated with m6A modification, similar to the 24.1% proportion of m6A-related genes among all the RNA modification genes. Moreover, these prognostic genes are predominantly classified as m6A writers, comprising 68.3% of candidate genes compared to 65.3% among all the RNA modification genes (Figure 2B). These findings imply that m6A modification may significantly influence tumorigenesis and progression across various cancers, with m6A writers playing a crucial role in regulating tumor progression.

We developed the RMS for 19 cancers (Figure S5A) and evaluated their predictive performance using ROC analysis (Figure 2C). The results show that the AUC for all the cancers significantly exceeds random chance (median AUC 0.66, range 0.56–0.92), suggesting that the RMS possesses strong prognostic predictive power across multiple cancers. Notably, PCPG (AUC = 0.92), THYM (AUC = 0.87), and READ (AUC = 0.83) exhibit particularly high predictive performance, with AUC values above 0.75, indicating promising clinical applications for the RMS in these cancers (Figure 2C). To further validate the predictive performance of the RMS, we constructed the model using external datasets obtained from the GEO database. Our analysis revealed that the RMS maintained robust predictive performance in ACC (AUC = 0.597), GBM (AUC = 0.620), and LUAD (AUC = 0.612) (Figure S5B). These results demonstrate the stable predictive efficacy and generalizability of our RMS across diverse cohorts.

The TMB is crucial in tumorigenesis and affects patient survival [43]. Our analysis revealed no significant correlation between the TMB and the RMS (Figure S5C), demonstrating that the RMS serves as an independent prognostic biomarker for patient outcomes independent of the TMB status.

We then analyzed the relationship between the risk RMS groups and the clinical survival time (Figure 2D; Table S7). Univariate Cox regression analysis revealed significant differences in survival between the risk RMS groups (with HR ≠ 1 in all cases). The Kaplan–Meier survival curves indicated that patients in the high-risk group generally had lower survival rates than those in the low-risk group (Figure S6A). In 17 out of 19 cancers (89%), the log-rank *p*-value was less than 0.05, except for KIRC and COAD, indicating statistically significant survival differences between the groups. Furthermore, validation using RMS data from five GEO cancer cohorts (Figure S6B) yielded results consistent with our initial findings in four cancers (HR ≠ 1). Overall, the RMS is closely associated with patient survival risk in most cancers, suggesting its potential as a prognostic indicator.

To explore the association between the RMS and clinical features, we analyzed the relationship between the RMS and the Stage as well as the TNM staging (Figure 2E and Figure S6C). Typically, cancers at Stages III and IV are considered advanced-stage and are linked to a poorer prognosis [43]. Our analysis revealed a significant correlation between the Stage and the risk RMS groups (Figure 2E). The high-risk group exhibited elevated proportions of advanced-stage cancers (Stage III/IV): 53.38% vs. 31.54% in KIRC (*p* = 1.989 × 10^−4^) and 27.70% vs. 15.16% in LUAD (*p* = 2.451 × 10^−5^). When validating the stage association in the independent GEO datasets, we observed a consistent trend toward an increased proportion of advanced-stage disease in high-risk KIRC patients (13.3% to 44.4%). The non-significant *p*-value (*p* = 0.15, Fisher’s exact test) likely reflects limited power (only four advanced-stage cases, *n* = 4) rather than evidence against a true effect. This observation warrants further validation in a larger cohort. The TNM parameters (T: tumor invasiveness, N: nodal spread, M: metastasis) further differentiated the risk RMS groups, with high-risk cohorts showing an increased metastatic burden (e.g., KIRC M1 incidence: 24.60% vs. 8.90%; *p* = 3.812 × 10^−3^). These findings establish the RMS as a genomic discriminator closely associated with clinical progression metrics, reinforcing its biological relevance and potential utility in stage-based clinical stratification.

To further investigate the gene expression differences between risk RMS groups, we identified DEGs ranging from 211 to 4958 per cancer (Figure 2F). ORA revealed significant enrichment of DEGs in the neuroactive ligand–receptor interaction pathway across all the cancers (Figure 2G). GSEA indicated that in the majority of cancers (73.7%), these genes were significantly enriched in the neuroactive ligand–receptor interaction pathway (Figure S7A,B; Table S8). Previous studies have shown that this pathway is often regulated by RNA modification regulators [44,45] and is closely associated with the TIME, particularly regarding immune suppression [46,47]. This suggests that the RMS may be linked to the TIME. In addition to the neuroactive ligand–receptor interaction pathway, other pathways exhibited cancer-specific enrichment patterns. For instance, the AMPK signaling pathway was significantly enriched only in BRCA during ORA and was most prominently enriched in BRCA according to GSEA; prior evidence suggests that this pathway plays a crucial role in tumor suppression in breast cancer [48] and is related to RNA modifications, such as FTO-dependent m6A demethylation, regulating lipid metabolism in skeletal muscle [49]. The thyroid hormone synthesis pathway showed significant enrichment in three cancers, including KIRP (Figure 2G). Thyroid hormones are known to interact with RNA modification regulators [50] and are critical modulators of cancer development and metastasis [51], as well as potential regulators in renal cell carcinoma [51].

### 3.3. Regulation of the TIME and Signaling Pathways by RMS

**Figure 3:**
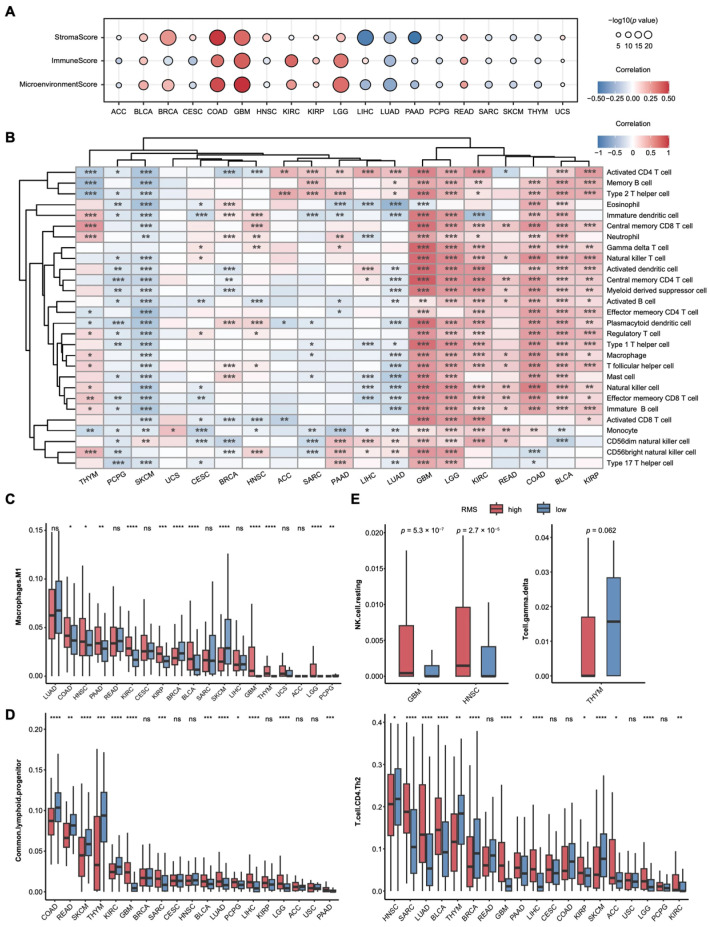
Integrative multi-algorithm analysis of the association between the RMS and the TIME. (**A**) Correlation analysis of RMS with ImmuneScore, StromalScore, and MicroenvironmentScore (quantified by xCell algorithm). (**B**) Spearman correlation heatmap between the RMS and the infiltration levels of 28 immune cell types (quantified by ssGSEA algorithm). Statistical significance was assessed using a two-tailed asymptotic *t*-test with Holm–Bonferroni correction. * *p* < 0.05, ** *p* < 0.01; *** *p* < 0.001. (**C**) Differences in immune infiltration levels of macrophages M1 cells (quantified by quanTIseq algorithm) between the risk RMS groups across 19 cancers (ns: not statistically significant). * *p* < 0.05, ** *p* < 0.01; *** *p* < 0.001, **** *p* < 0.0001. (**D**) Differences in the immune infiltration levels of Common.lymphoid.progenitor (left) and T.cell.CD4.Th2 cells (right) (from TIMER database) between the risk RMS groups across 19 cancers (ns: not statistically significant). * *p* < 0.05, ** *p* < 0.01; *** *p* < 0.001, **** *p* < 0.0001. (**E**) Differences in the immune infiltration levels of NK.cell.resting cells in GBM and HNSC (**left**), and T.cell.gamma.delta cells in THYM (**right**) (from TIMER database) between the risk RMS groups.

To explore the relationship between the RMS and the TIME, we first evaluated the proportions of various cell types within the TIME. We assessed the ImmuneScore, StromalScore, and MicroenvironmentScore infiltration levels across 19 cancers. Their correlations with the RMS indicate that in GBM and COAD, the RMS is significantly positively correlated with all three scores (Figure 3A). Across five GEO cancer datasets, GBM consistently showed strong positive associations between the RMS and all three scoring metrics, reinforcing its biological and clinical relevance (Figure S8A).

Next, we quantified the infiltration levels of 28 different immune cell types in tumor tissues and assessed their correlation with the RMS. The analysis revealed a biphasic distribution in the correlation: in some cancers, the RMS is negatively correlated with immune cell infiltration, while in others, it is positively correlated (Figure 3B). Notably, in GBM and LGG, nearly all the immune cell infiltrates (27 of 28, 96.4%) positively correlated with the RMS. In the GBM cohort of the four GEO cancer datasets, most immune cell populations (22 of 28, 78.6%) showed significant positive correlations with the RMS, consistent with the above findings and underscoring its potential to capture the distinctive immune landscape of GBM (Figure S8B). This finding aligns with previous studies [52,53] and suggests that the RMS may delineate cancer-specific immune infiltration patterns, offering potential insights for immunotherapeutic strategies in these tumors.

We next estimated the proportions of immune cells in the tumor immune microenvironment (TIME) using transcriptome data. This analysis quantified the infiltration of 10 immune cell types across 19 cancers and revealed significant differences between the RMS risk groups (Figure 3C; Table S9). Consistent trends were observed in five independent GEO cohorts, with GBM showing the strongest association. Similar patterns were also detected in ACC and LUAD, aligning with the TCGA results (Figure S8C).

The differences in M1 macrophage infiltration were especially prominent in GBM and THYM. To elucidate the underlying molecular mechanisms, we conducted differential transcriptome analysis between the RMS high-risk and low-risk groups, followed by enrichment analyses using both KEGG pathway over-representation analysis and GSEA (Figure S8D,E). The M1 macrophage-associated pathways were consistently enriched across 19 cancers, encompassing pro-inflammatory and immune-regulatory processes, including the NF-κB pathway (cytokine regulation) [54], JAK-STAT signaling (STAT1-mediated M1 polarization) [55], NOD-like receptor signaling (inflammasome activation) [56], and IL-17 signaling (inflammation promotion) [57]. Metabolic reprogramming signatures were also evident, such as glycolysis/gluconeogenesis [58] and PPAR signaling (negative regulation of polarization) [59], along with enrichment in cell adhesion molecules [60]. GSEA further confirmed the coordinated up- or down-regulation of these pathways across cancers, underscoring the potential of M1 macrophages as therapeutic targets.

To validate these associations, we integrated the immune infiltration profiles from multiple algorithms in the TIMER database (Figure 3D,E; Table S10). Common lymphoid progenitors exhibited significant differences between the RMS risk groups in 13 cancers, with LUAD showing consistent results in the GEO datasets (Figure S8F). Although GBM did not reach statistical significance (*p* > 0.05), its high-risk group displayed a trend toward increased immune infiltration. Th2 cells showed marked differences in 14 cancers and across four GEO cohorts, further linking the RMS to immune cell recruitment (Figure S8F). Resting NK cells demonstrated cancer-specific patterns in HNSC and GBM [61,62], while γδ T cells were selectively enriched in THYM (Figure 3E; Table S10), consistent with their reported antitumor roles [63].

Collectively, these findings establish a robust association between the RMS and the composition of the TIME, suggesting that the RMS may influence immune cell recruitment and function across diverse cancers, with implications for prognostication and immunotherapy development.

**Figure 4:**
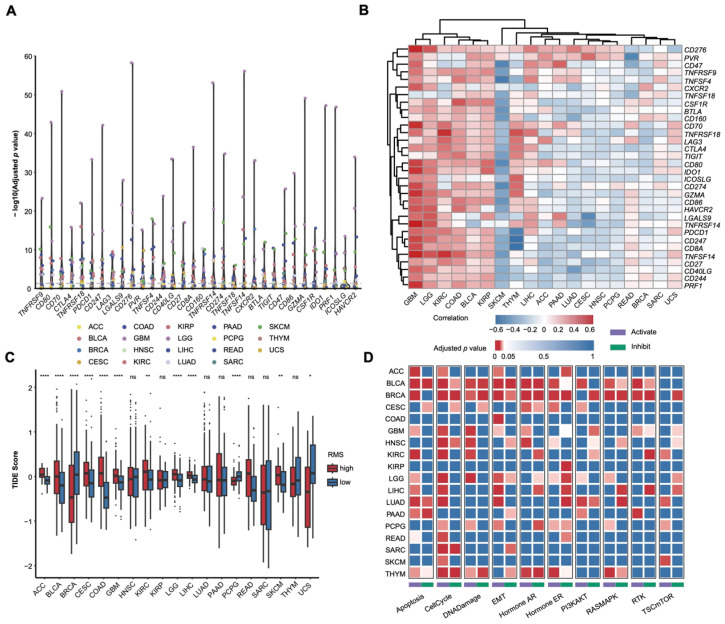
Potential roles of the RMS in tumor immunotherapy and signaling pathways. (**A**) Differential analysis of immune checkpoint molecules between the risk RMS groups across 19 cancers (Wilcoxon test; dashed line indicates adjusted *p* value < 0.05). (**B**) Spearman correlation heatmap between the RMS and the expression levels of immune checkpoint molecules. (**C**) Comparison of the TIDE scores between the risk RMS groups (Wilcoxon test; ns: not statistically significant). * *p* < 0.05, ** *p* < 0.01, **** *p* < 0.0001. (**D**) GSCA signaling pathway analysis between risk RMS groups in 18 cancers.

Immune checkpoint molecules are crucial in tumor immune evasion and serve as important targets for immunotherapy. We further analyzed the expression levels of 32 immune checkpoint molecules across the risk RMS groups (Figure 4A). The results indicate significant differences in the checkpoint expression levels between the two RMS groups in most cancers (*p* < 0.05), with the most pronounced effects observed in GBM (*n* = 31), which is fully consistent with the GEO external data validation (Figure S9A). Notably, *TNFRSF18*, *LAG3*, and *PVR* exhibit significant differences in 13 to 15 cancers. The Spearman correlation analysis revealed a biphasic correlation pattern between the RMS and the checkpoints’ expression: in GBM, the RMS is almost uniformly positively correlated with immune checkpoint expression, while in SKCM, the RMS is almost uniformly negatively correlated (Figure 4B). The GEO data validation again corresponded to the results (Figure S9B). This suggests that the RMS may regulate the TIME by influencing immune checkpoint molecule expression, with effects that vary by cancer type.

The TIDE significantly impacts the outcomes of immunotherapy. To predict the efficacy of immunotherapy, we compared the TIDE scores between the risk RMS groups. Consistent with our expectations, in nine cancers (75% of those with significant differences), the high-risk RMS group exhibits higher TIDE scores (Figure 4C). Prior research has demonstrated that inhibiting RNA modification enzymes (e.g., through ALKBH5/FTO inhibitors or METTL3/14 knockdown) can significantly enhance the antitumor effects of PD-L1 blockade [64]. This finding aligns with our result, suggesting that increased RNA modification activity may bolster tumor immune evasion while compromising immunotherapy efficacy. Notably, the RMS is significantly elevated in ACC compared to others (Figure S5A), leading us to speculate that RNA modifications may enhance immune evasion in ACC. Recent studies have underscored the potential of epigenetic regulation in promoting immune evasion in this subtype [65]. Interestingly, patients in the high-risk RMS group exhibit lower TIDE scores in BRCA and PCPG, implying that immune checkpoint inhibitors (ICIs) may be more effective for these patients [66]. Overall, these results indicate that RNA modifications may reshape the TIME, thereby influencing the clinical efficacy of ICIs.

To further explore the impact of the RMS on tumor cell signaling pathways, we assessed the activation or suppression of various signaling pathways among the DEGs between the risk RMS groups. The results reveal that in 14 cancers (77.8%), the cell cycle pathway is significantly activated (Figure 4D). Given that the mammalian cell cycle is a tightly regulated process, aberrant activation can lead to uncontrolled genomic replication and cell division, which are closely associated with cancer’s uncontrolled proliferation—a finding aligned with established cancer characteristics [67]. Notably, in colon adenocarcinoma (COAD), only the epithelial–mesenchymal transition (EMT) pathway is significantly activated (*p* = 1.0495 × 10^−5^). The EMT pathway is crucial for the progression and metastasis of COAD [68], suggesting that the RMS may facilitate the malignant progression of COAD through EMT pathway activation. In summary, the RMS appears to influence key signaling pathways, such as the cell cycle, in a cancer-specific manner, indicating its significant regulatory role in tumorigenesis and progression.

### 3.4. Drug Sensitivity Analysis Based on RMS and Candidate Drug Screening

**Figure 5:**
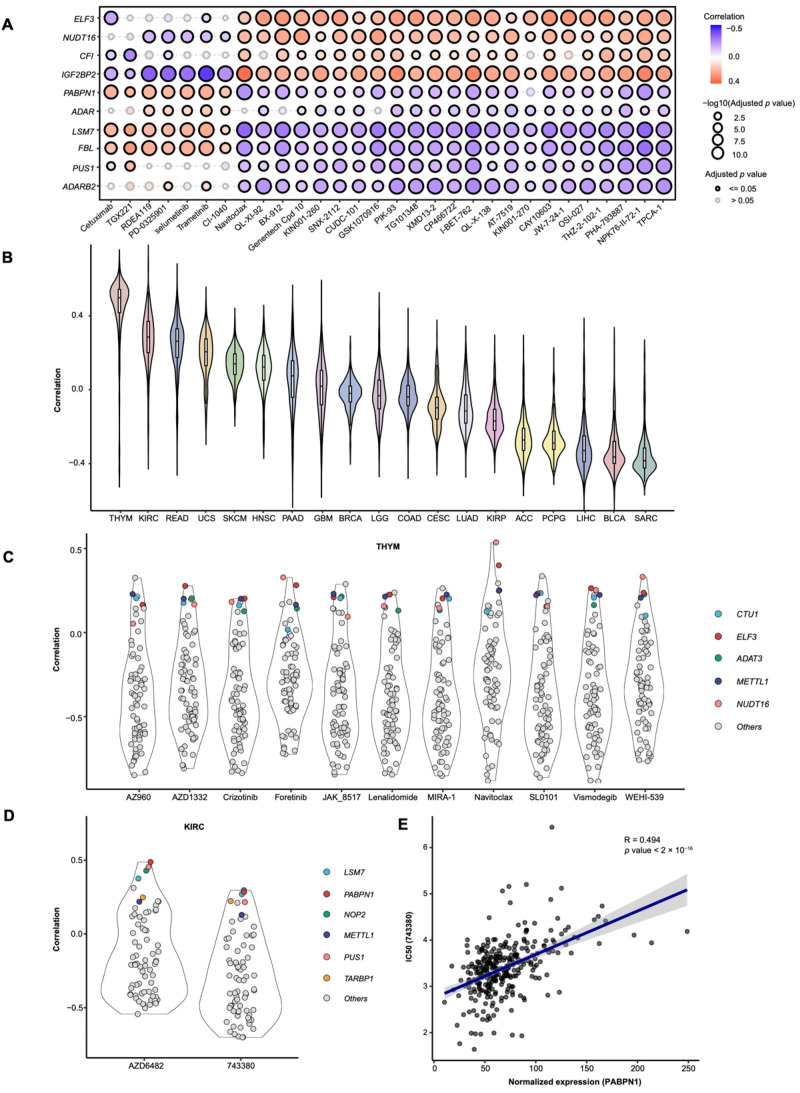
Drug sensitivity analysis of cancers highly correlated with the RMS. (**A**) Top 30 drugs potentially linked to genes (*ELF3*, *NUDT16*, *CFI*, *IGF2BP2*, *PABPN1*, *ADAR*, *LSM7*, *FBL*, *PUS1*, *ADARB2*) via GSCALite analysis. (**B**) Spearman correlation between the RMS and the predicted IC50 values of drugs across cancer types. (**C**) Distribution plot of the Spearman correlations between drugs (with RMS-IC50 correlation > 0.6) and RNA modification genes in THYM. (**D**) Distribution plot of the Spearman correlations between drugs (with RMS-IC50 correlation > 0.6) and RNA modification genes in KIRC. (**E**) Spearman correlation between the IC50 values of drug 743,380 (oncrasin-72) and *PABPN1* expression.

To evaluate the relationship between RMS-related genes and drug sensitivity, as well as to identify novel candidate drugs, we conducted a drug sensitivity analysis of RMS-related genes (Figure 5A and Figure S10). The results indicate that *ELF3*, *NUDT16*, *CFI*, and *IGF2BP2* are positively correlated with the majority of drugs, suggesting that higher expression of these genes may contribute to drug resistance. Conversely, genes such as *PABPN1*, *LSM7*, and *FBL* are negatively correlated with most drugs, implying that increased expression may confer drug sensitivity.

We predicted the IC50 values for 285 drugs based on the gene expression matrix from samples of the risk RMS groups and conducted a correlation analysis (Table S11). The results show that in THYM and KIRC, the correlation between the RMS levels and the drug IC50 values is generally high, with average correlation coefficients of 0.4 for THYM and 0.2 for KIRC (Figure 5B). This suggests that the RMS may influence cancer cell sensitivity to multiple drugs.

To further identify potential candidate drugs, we extracted those with a high correlation (|correlation coefficient| > 0.6) and analyzed the relationship between their IC50 values and RNA modification genes. The results indicate that in THYM, genes such as *ELF3*, *NUDT16*, and *FBL* exhibit high correlation levels with the selected drugs (average correlation coefficient > 0.2; Figure 5C). In contrast, in KIRC, *PABPN1* and *LSM7* show higher correlation levels with the selected drugs (average correlation coefficient > 0.3; Figure 5D,E). Previous studies have demonstrated that *PABPN1* significantly influences the progression of KIRC [69], suggesting that targeting *PABPN1* may offer a new therapeutic strategy for this cancer. Additionally, we identified the drug 743,380 (1-[(3-chlorophenyl) methyl]-1H-indole-3-methanol, oncrasin-72) as a potential anticancer agent. This compound may induce antitumor activity by modulating multiple cancer-related pathways, making it a promising candidate for treating solid tumors such as lung, colon, ovarian, kidney, and breast cancers [70].

### 3.5. Single-Cell and Spatial Transcriptomic Analyses Reveal Cell-Type-Specific Expression of RMS-Related Genes

Given the distinct CNV profiles and immune characteristics of GBM and SKCM, we conducted an integrated analysis combining single-cell sequencing (Figure S7A), spatial transcriptomics (Figure S7B), and immunohistochemical validation (Table S12) to systematically evaluate the biological significance and clinical applicability of the RMS in these two cancer types.

**Figure 6:**
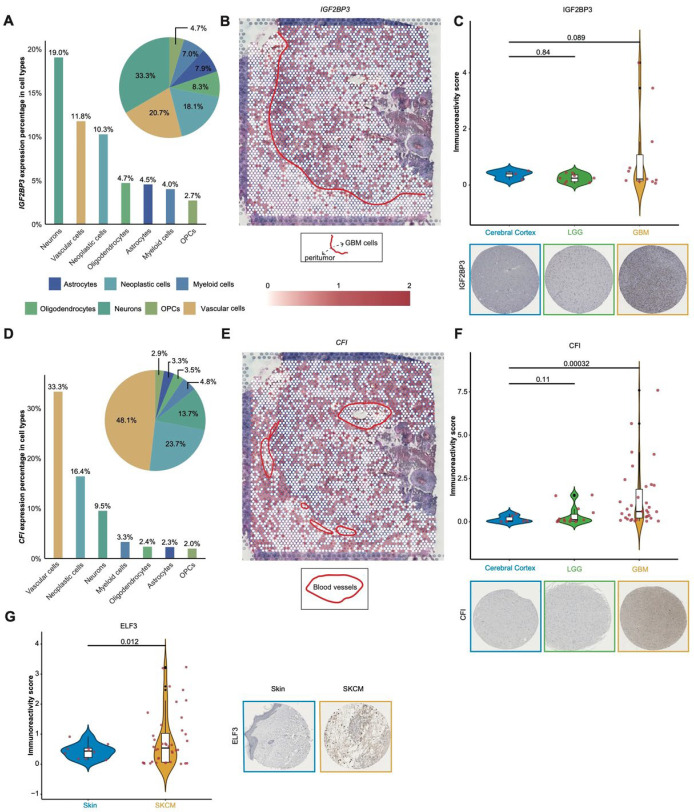
Single-cell, spatial transcriptomic, and immunohistochemical analyses of *IGF2BP3*, *CFI*, and *ELF3* in GBM and SKCM. (**A**) Distribution of *IGF2BP3* across cell clusters in GBM via single-cell RNA sequencing. The inserted pie plot displays the relative distribution ratio. (**B**) Spatial transcriptomic mapping reveals the *IGF2BP3* expression distribution across the GBM specimen. (**C**) Immunohistochemistry slides and quantitative immunoreactivity scores for the IGF2BP3 protein are shown, with statistical significance evaluated by a one-tailed Student’s *t*-test. (**D**) Distribution of *CFI* across cell clusters in GBM via single-cell RNA sequencing. The inserted pie plot displays the relative distribution ratio. (**E**) Spatial transcriptomic mapping reveals the *CFI* expression distribution across the GBM specimen. (**F**) Immunohistochemistry slides and quantitative immunoreactivity scores for the CFI protein are shown, with statistical significance evaluated by a one-tailed Student’s *t*-test. (**G**) Immunohistochemistry slides and quantitative immunoreactivity scores for the ELF3 protein, with statistical significance evaluated by a one-tailed Student’s *t*-test.

IGF2BP3, an m6A “reader” regulating RNA metabolism (Figure S2A,B), exhibits pan-cancer amplification (Figure 1D), functions as a core RMS element (Figure 2A), and critically influences GBM and multi-cancer prognosis. Single-cell transcriptomic analysis reveals a pronounced cell-type-specific expression pattern of *IGF2BP3* in GBM, with enrichment primarily in neurons (19.0%), vascular cells (11.8%), and neoplastic cells (10.3%) (Figure 6A and Figure S7C). Spatial transcriptomic analysis demonstrates that *IGF2BP3* expression is concentrated in the infiltrating front and perivascular regions (Figure 6B and Figure S7B), which may facilitate local tumor cell invasion and dissemination and is associated with a poor prognosis. Previous studies have shown that *IGF2BP3* contributes to adverse outcomes by promoting GBM-specific proliferation and invasion [71,72], a conclusion supported by our findings. Immunohistochemical staining indicates that a subset of GBM samples exhibit high IGF2BP3 protein expression, while LGG samples consistently show negative staining (Figure 6C). These results elucidate the mechanisms by which *IGF2BP3* contributes to GBM progression and support its role as an important molecular marker for glioma grading and diagnosis.

CFI, an APA “writer” gene, plays a critical role in the RMS (Figure 2A) and shows drug sensitivity correlations (Figure 5A and Figure S10), with distinctive expression patterns in pan-cancer analyses (Figure 1A and Figure S2A,B). The single-cell transcriptomic results demonstrate that *CFI* is predominantly expressed in vascular cells (33.3%), followed by neoplastic cells (16.4%) and neurons (9.5%) (Figure 6D). Spatial transcriptomic analysis indicates that *CFI* is diffusely distributed in the TIME, with increased expression around blood vessels (Figure 6E and Figure S11B), suggesting a vital role in GBM vascular biology. Immunohistochemical staining corroborates these findings, showing significantly higher CFI protein expression in GBM compared to normal cerebral cortex and LGG (Figure 6F). As a core regulatory molecule of the complement system, *CFI* is known to participate in immune regulation of normal vasculature by inhibiting complement activation and modulating endothelial inflammatory responses to maintain vascular homeostasis [73]. While previous studies have noted a role of *CFI* in GBM [74], its specific function in tumor-associated vascular hyperplasia remains unclear. These findings suggest *CFI* may regulate GBM vasculature through potential mechanisms such as angiogenesis modulation or vascular microenvironment remodeling (though the precise mechanisms remain uncharacterized), positioning it as a candidate anti-angiogenic target.

ELF3, an m6A “reader” with cancer-specific expression/mutation patterns (Figure 1A–C and Figure S2A,B), demonstrates drug sensitivity correlations (Figure 5A and Figure S10), suggesting dual roles in the therapeutic response and tumor progression. Immunohistochemical analysis reveals that ELF3 protein expression is significantly elevated in SKCM tissue compared to normal skin (*p* = 0.012, Figure 6G) and is closely associated with a poor prognosis in SKCM patients [75], suggesting that ELF3 is functionally significant in SKCM development. While previous studies have investigated m6A modifications in melanoma, the specific biological role of *ELF3* as an m6A “reader” in SKCM remains poorly understood. ELF3 may mediate SKCM pathogenesis and drug resistance through undefined mechanisms, offering potential diagnostic and therapeutic targets.

## 4. Discussion

This study establishes, for the first time, a pan-cancer RMS by integrating multimodal data from 33 cancer types, encompassing 15 types of RNA modifications. This model systematically reveals the multidimensional features of the RNA modification genes regarding the genomic variation, epigenetic regulation, and clinical prognosis. The application of single-cell and spatial transcriptomics overcomes the resolution limitations of conventional transcriptome analyses, enabling precise delineation of the spatial heterogeneity in gene expression, while immunohistochemical quantification directly links molecular mechanisms to pathological phenotypes, providing multidimensional evidence to support the clinical translation of RNA modification research.

These findings expand the pan-cancer research dimension of RNA modifications and underscore the significance of a multi-modification interaction network. Unlike previous studies focusing on a single modification type (e.g., m6A) [76], our study systematically analyzes the commonalities and heterogeneities of 15 RNA modification types across various cancers, thereby addressing a critical gap in the multi-modification interaction research. For example, the “cross-regulatory” mechanism through which ALKBH1 simultaneously regulates both m5C and m1A [77] indicates that the complexity of the epitranscriptomic network may greatly exceed our current understanding. Clinically, the RMS model overcomes the limitations of individual cancer prognostic markers, such as the independent predictive value of *IGF2BP3* in glioma [42]. Its significant association with TNM staging—evidenced by the differences in the M1 stage proportions in KIRC (Figure 2E)—enhances the biological interpretability of the staging system. Notably, the RMS score in ACC is the highest among all the cancer types (Figure 5A), and the TIDE score in the risk RMS groups is significantly elevated (Figure 4C). This suggests that aberrant RNA modification activity may diminish the immunotherapy response by promoting immune evasion. This observation aligns with recent reports indicating that epigenetic regulation fosters immune suppression in ACC [65]. These findings reinforce the potential of the RMS as a crucial indicator for evaluating immune evasion in ACC. Overall, this research provides new evidence for RNA modification-mediated regulation of the TIME and establishes a foundation for the clinical translation of combined RNA modification inhibitors and immune checkpoint blockade therapies.

The results indicate that the interplay between the RNA modifications and the TIME may represent a fundamental mechanism driving tumor progression. The strong correlations between the RMS and immune checkpoint molecules such as *TNFRSF9* and *LAG3* (Figure 4A,B), suggest that RNA modifications could serve as novel targets to prevent immune evasion. For instance, inhibitors targeting ALKBH5/FTO, or the loss of *METTL3/14*, have demonstrated the enhanced efficacy of PD-L1 blockade by upregulating immune checkpoint expression and promoting cytokine secretion [23,78,79]. Additionally, the increased M1 macrophage infiltration (in GBM and THYM) and γδ T-cell enrichment (in THYM) within the high-risk RMS group (Figure 3C–E) indicate that RNA modifications might reshape the TIME by modulating immune cell recruitment or functional states [63]. This phenomenon may be closely related to the functional properties of RNA modification regulators. Our data indicate that m6A writers dominate the RNA modification regulatory network (Figure 2B). This dominance not only highlights their central role in the modification network but also suggests that their dysregulation could significantly impact tumor progression [80,81]. For example, *METTL3*, as an m6A writer, significantly influences multiple cancers by dynamically regulating immune cell infiltration [22,29]. In contrast, the functions of readers and erasers may depend more on specific targets or microenvironmental contexts, resulting in relatively limited pan-cancer prognostic value [82].

Based on these mechanistic features, the RMS model presents a three-stage clinical translation pathway: prognostic stratification, treatment optimization, and precision diagnosis. As a prognostic stratification tool, it complements traditional TNM staging (Figure 2E) by identifying high-risk patients, such as those with elevated M1 proportions in KIRC, and guiding individualized follow-up strategies. In terms of treatment optimization, the lower TIDE scores in the high-risk RMS group in certain cancers (e.g., BRCA and PCPG) suggest that immune checkpoint inhibitors (ICIs) may be more effective for this subgroup. The candidate drugs identified through the drug sensitivity analysis (e.g., oncrasin-72) provide a foundation for combination therapies (Figure 5C–E). Oncrasin-72 exerts its activity by inhibiting C-terminal domain phosphorylation of RNA polymerase II in sensitive human cancer cells, thereby activating JNK, suppressing JAK2/STAT3 phosphorylation, and reducing cyclin D1 expression. Its antitumor effect is partially attenuated by blocking either constitutively active STAT3 or JNK activation [70]. Additional studies identify oncrasin-72 as a novel STAT3 inhibitor that (i) suppresses proliferation across the NCI-60 cell line panel from diverse tissue origins, (ii) induces dose-dependent tumor regression in xenograft models, and (iii) significantly inhibits both tumor growth and STAT3 phosphorylation (*p*-STAT3) in lung cancer models. Mechanistically, oncrasin-72 promotes apoptosis in lung cancer via ROS generation following STAT3 inhibition [83]. Furthermore, the spatial expression patterns of *IGF2BP3* and *CFI* in GBM (Figure 6A–F) may translate into radiomic features, facilitating noninvasive diagnosis [84]. These findings offer new avenues for the clinical translation of RNA modification-related biomarkers and the development of precision treatment strategies.

The expression data in this study were derived from the UCSC Xena platform, which provides uniformly processed and batch-corrected TCGA and GTEx datasets. Nevertheless, batch effects remain an inherent challenge in multi-platform data integration and may potentially influence downstream analyses. Therefore, future studies incorporating more diverse datasets should continue to rigorously assess and mitigate the batch effects to ensure the robustness of the findings. It is also important to acknowledge the methodological limitations impacting the clinical translation potential of this study. In terms of the data coverage, the limited sample size for some rare cancers (e.g., LAML/UVM) may restrict the generalizability of the RMS model, highlighting the need for validation in larger, multicenter cohorts to confirm its pan-cancer applicability. At the technical resolution level, analyses based on bulk transcriptomic data cannot capture the dynamic heterogeneity of RNA modifications at the single-cell level. Therefore, future studies should integrate single-cell RNA sequencing (scRNA-seq) with modification-specific sequencing techniques (e.g., m6A-CLIP) to characterize the modification profiles of specific cell subpopulations within the microenvironment. Regarding the depth of the mechanistic validation, the protumor mechanisms of key genes such as *IGF2BP3* and *CFI* warrant further investigation using organoid models or gene editing to validate their functions in three-dimensional contexts [85,86]. To construct a comprehensive overview of the RNA modification regulatory network, future research should integrate epigenomic data (e.g., ATAC-seq) and proteomic data (e.g., mass spectrometry) to elucidate the epigenetic regulatory pathways of modification factors and their downstream effector proteins. Additionally, prospective clinical cohort studies should be conducted to evaluate the RMS model’s potential in guiding personalized treatment strategies. However, the practical implementation of the RMS model in clinical settings faces several challenges. These include the need for standardized, cost-effective assays for RNA modification profiling, integration with existing diagnostic workflows, and validation across diverse patient populations to ensure reproducibility and clinical utility. Addressing these issues will be crucial for translating the RMS model from research into routine oncology practice.

## 5. Conclusions

This study systematically elucidates the multilayered regulatory roles of RNA modifications in tumorigenesis and progression, highlighting their functions and regulatory factors in tumor cell signaling pathways, TIME remodeling, and drug sensitivity. The novel findings regarding candidate genes such as *CFI* and *ELF3* not only enhance our understanding of tumor biology but also pave the way for precision diagnosis and targeted therapy. This will strengthen the clinical translation of RNA modification-related discoveries and provide a more robust theoretical and practical foundation for precision oncology.

## Data Availability

The datasets used and/or analyzed during the current study are available from the corresponding author on reasonable request.

## Supplementary Materials

The following supporting information can be downloaded at https://www.mdpi.com/article/10.3390/cancers17162695/s1. Figure S1. Pan-cancer landscape of RNA modifications and their regulatory genes. Figure S2. Expression profiles of RNA modification genes. Figure S3. Genomic alterations of RNA modification genes. Figure S4. Methylation profiles of RNA modification genes. Figure S5. RMS construction and ROC verification. Figure S6. Clinical relevance of RMS. Figure S7. Pathway enrichment analysis of RMS. Figure S8. Comprehensive multi-algorithm analysis of the correlation between RMS and TIME. Figure S9. Potential roles of RMS in tumor immunotherapy in GEO datasets. Figure S10. Sensitivity analysis of RMS-related drug. Figure S11. Single-cell and spatial transcriptomic expression analyses in GBM. Table S1. Overview of published studies on RNA modifications in cancer. Table S2. Systematic annotation of 15 major RNA modification types with associated regulatory genes curated from published studies. Table S3. Comprehensive classification of RNA modification regulators across 15 major modification types. Table S4. Pan-cancer landscape of mutation frequencies in RNA modification regulators. Table S5. Pan-cancer CNV landscape of RNA modification regulators. Table S6. Integrated results of DESeq2 differential expression analysis and LASSO regression selection. Table S7. Survival analysis: log-rank p-values, Hazard Ratios (HR), and 95% Confidence Intervals (CI). Table S8. GSEA of KEGG pathways stratified by RMS risk groups. Table S9. Assessment of immune infiltration levels using QuanTIseq. Table S10. Assessment of immune infiltration levels using data from the TIMER database. Table S11. Summary of predicted IC50 values for various drugs. Table S12. Quantification of the intensity of immunohistochemical staining.
