# Supplementary material for: Unraveling the Clinical Landscape of RNA Modification Regulators with Multi-Omics Insights in Pan-Cancer
Source: Cancers (Basel). 2025 Aug 19;17(16):2695. doi: 10.3390/cancers17162695 (PMC12384070; doi:10.3390/cancers17162695)
Supplement: Supplementary file 1 [file cancers-17-02695-s001.zip › SupplementalFigures.pdf]

Supplementary Information for

Unraveling the Clinical Landscape of RNA Modification Regulators with Multi-Omics Insights in Pan-Cancer

This PDF file includes:

Supplementary Figures S1 to S11

Supplementary figures:

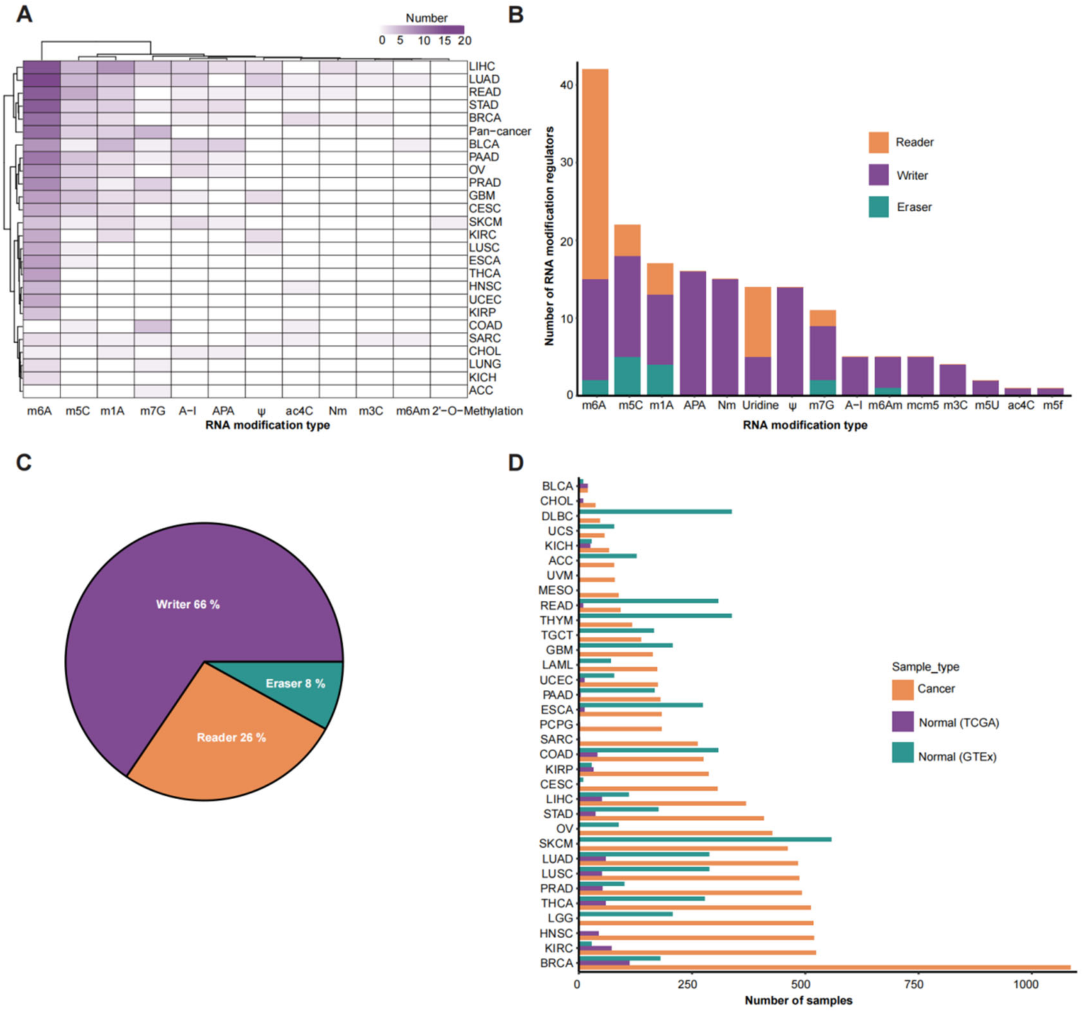

Figure. S1. Pan-cancer landscape of RNA modifications and their regulatory genes. (A) Review of current studies on RNA modifications in cancer (B) Bar plot of 170 RNA modification regulatory genes across 15 RNA modification types (C) Pie chart of functional categories for 170 RNA modification regulatory genes (D) Bar plot of sample counts for 33 cancers from TCGA and GTEx databases.

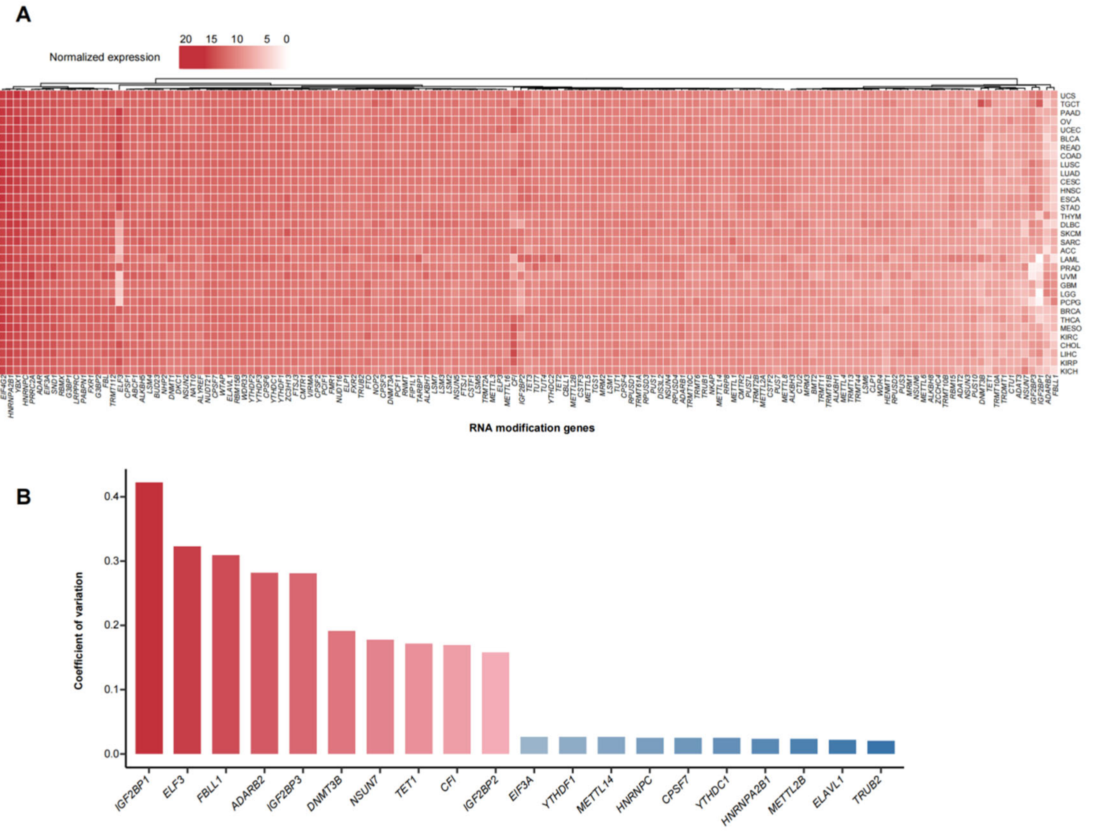

Figure. S2. Expression profiles of RNA modification genes.

(A) Expression profiles of RNA modification regulatory genes across 33 cancer types (unit: TPM)

(B) Coefficient of variation (CV = standard deviation/mean) of gene expression across cancers (red: CV top 10 genes, blue: CV bottom 10 genes).

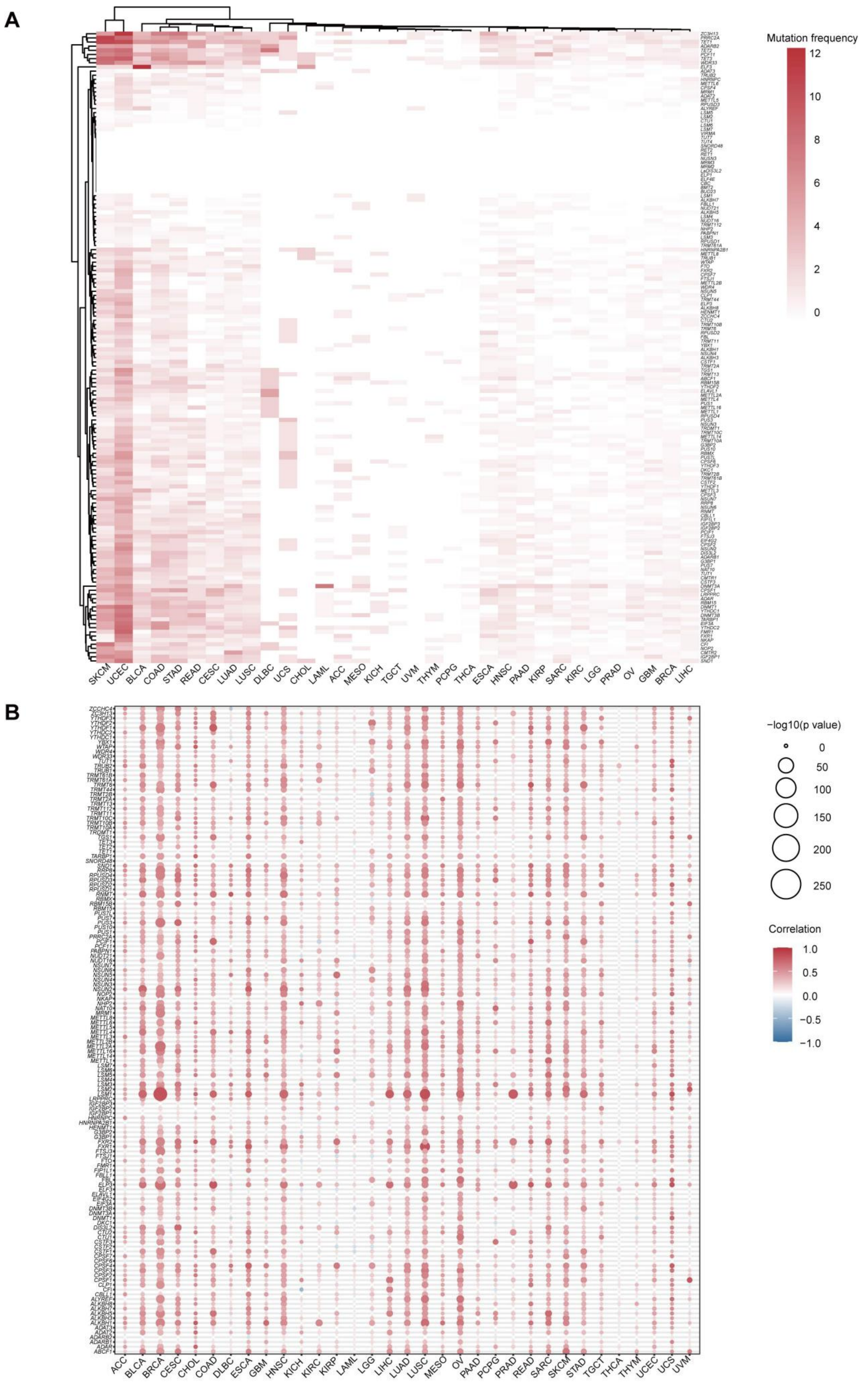

Figure. S3. Genomic alterations of RNA modification genes.

(A) Heatmap of mutation frequencies (×100) for RNA modification regulatory genes across 33 cancer types (B) Bubble plot showing correlations between CNV and expression levels of RNA modification genes in 33 cancers.

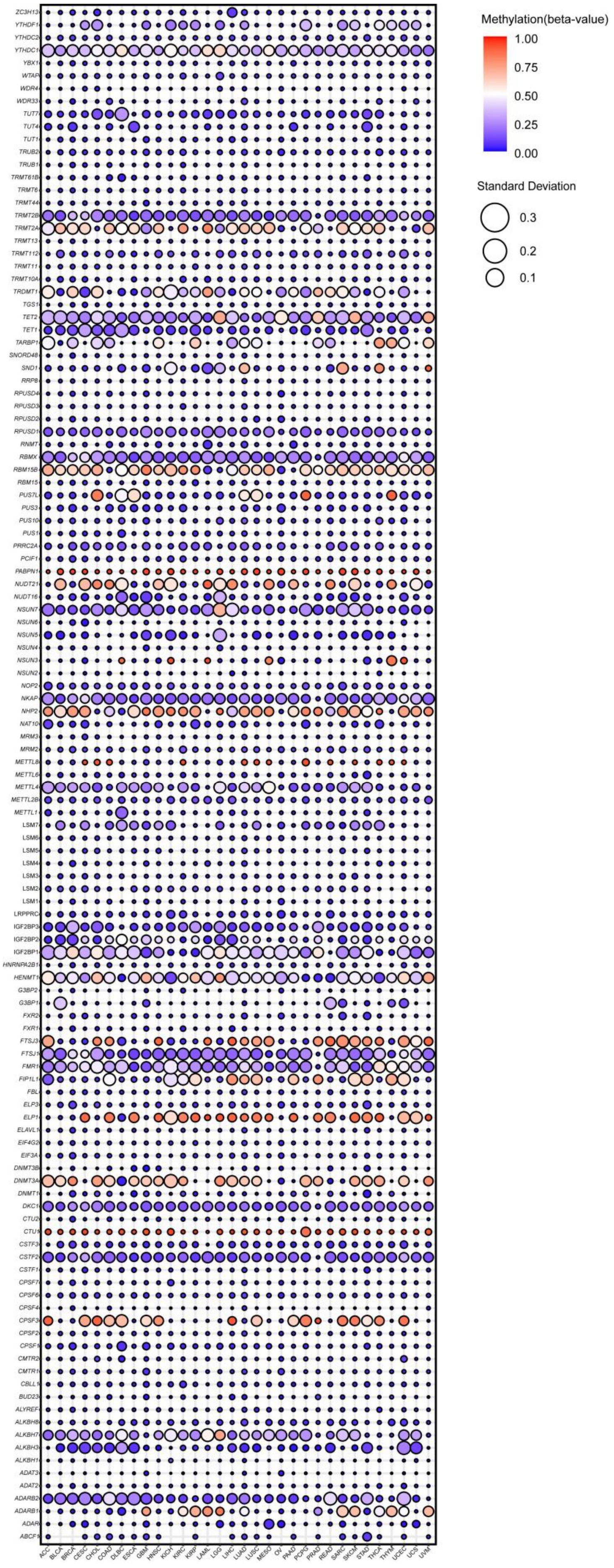

ZC3H13
YTHDF1
YTHDC2
YTHDC1
YBX1
WTAP
WDR4
WDR33
TUT7
TUT4
TUT1
TRUB2
TRUB1
TRMT61B
TRMT6
TRMT44
TRMT2B
TRMT2A
TRMT13
TRMT112
TRMT11
TRMT10A
TRDMT1
TGS1
TET2
TET1
TARBP1
SNORD48
SND1
RRP8
RPUSD4
RPUSD3
RPUSD2
RPUSD1
RNMT
RBMX
RBM15B
RBM15
PUS7L
PUS3
PUS10
PUS1
PRRC2A
PCIF1
PABPN1
NUDT21
NUDT16
NSUN7
NSUN6
NSUN5
NSUN4
NSUN3
NSUN2
NOP2
NKAP
NHP2
NAT10
MRM3
MRM2
METTL8
METTL6
METTL4
METTL2B
METTL1
LSM7
LSM6
LSM5
LSM4
LSM3
LSM2
LSM1
LRPPRC
IGF2BP3
IGF2BP2
IGF2BP1
HNRNPA2B1
HENMT1
G3BP2
G3BP1
FXR2
FXR1
FTSJ3
FTSJ1
FMR1
FIP1L1
FBL
ELP3
ELP1
ELAVL1
EIF4G2
EIF3A
DNMT3B
DNMT3A
DNMT1
DKC1
CTU2
CTU1
CSTF3
CSTF2
CSTF1
CPSF7
CPSF6
CPSF4
CPSF3
CPSF2
CPSF1
CMTR2
CMTR1
CBLL1
BUD23
ALYREF
ALKBH8
ALKBH7
ALKBH3
ALKBH1
ADAT3
ADAT2
ADARB2
ADARB1
ADAR
ABCF1
ACC
BLCA
BRCA
CESC
CHOL
COAD
DLBC
ESCA
GBM
HNSC
KICH
KIRC
KIRP
LAML
LGG
LIHC
LUAD
LUSC
MESO
OV
PAAD
PCPG
PRAD
READ
SARC
SKCM
STAD
THCA
THYM
UCEC
UCS
UVM
Methylation(beta-value)
1.00
0.75
0.50
0.25
0.00
Standard Deviation
0.3
0.2
0.1

Figure. S4. Methylation profiles of RNA modification genes.

Methylation levels (beta-value) and their standard deviation of RNA modification genes.

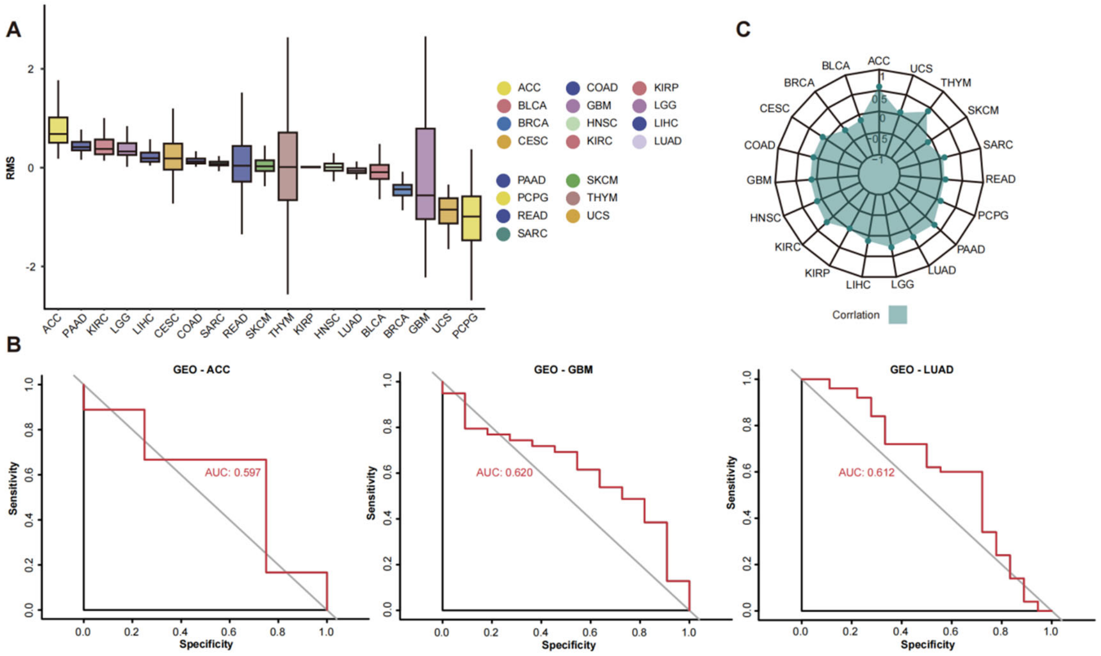

Figure. S5. RMS construction and ROC verification.

(A) The RMS score across 19 cancers based on key genes (B) Area under the receiver operating characteristic curve (AUC) of the RMS in ACC, GBM, and LUAD, evaluated using GEO datasets (C) Radar plot of the correlation between RMS and TMB.

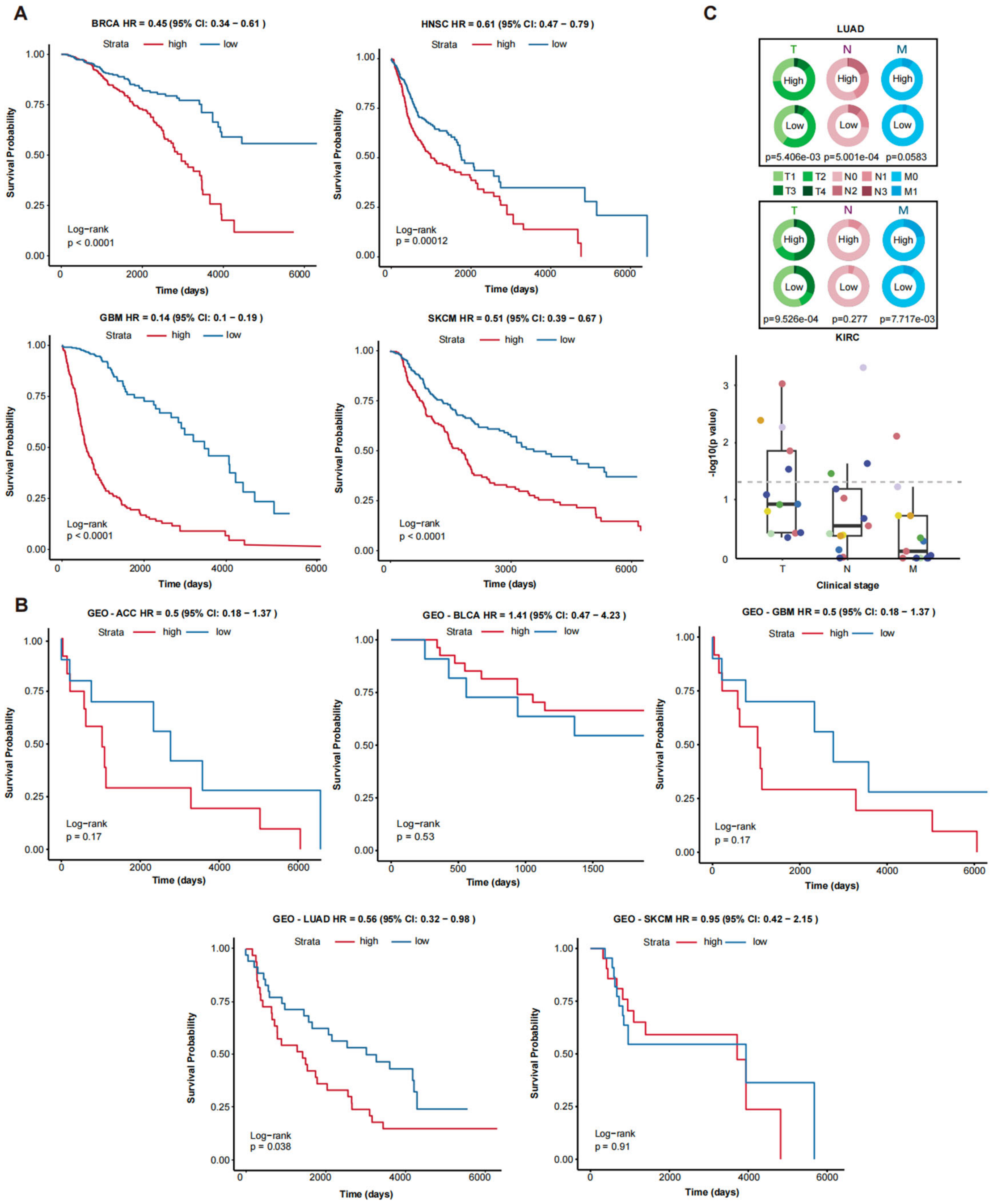

Figure. S6. Clinical relevance of RMS.

(A) Kaplan-Meier survival curves comparing risk RMS groups (stratified by median RMS) in BRCA, HNSC, GBM, and SKCM (B) Survival analysis validation of the GEO datasets. Kaplan-Meier survival curves comparing risk RMS groups (stratified by median RMS) in ACC, BLCA, GBM, LUAD, and SKCM (C) Chi-square test of the association between clinical TNM stage and risk RMS groups (bottom), with significant correlation highlighted in LUAD and KIRC (top).

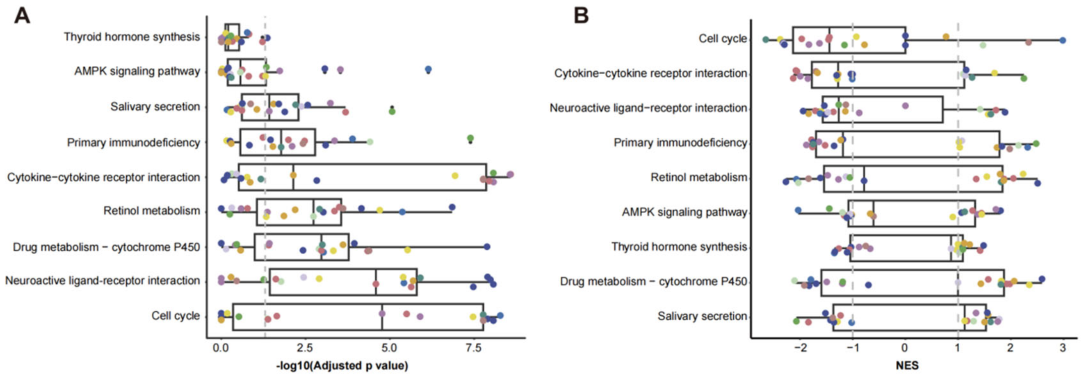

Figure. S7. Pathway enrichment analysis of RMS.

(A) Gene Set Enrichment Analysis (GSEA) comparing risk RMS groups across 19 cancers, showing adjusted p values (B) Gene Set Enrichment Analysis (GSEA) comparing risk RMS groups across 19 cancers, showing normalized enrichment scores (NES).

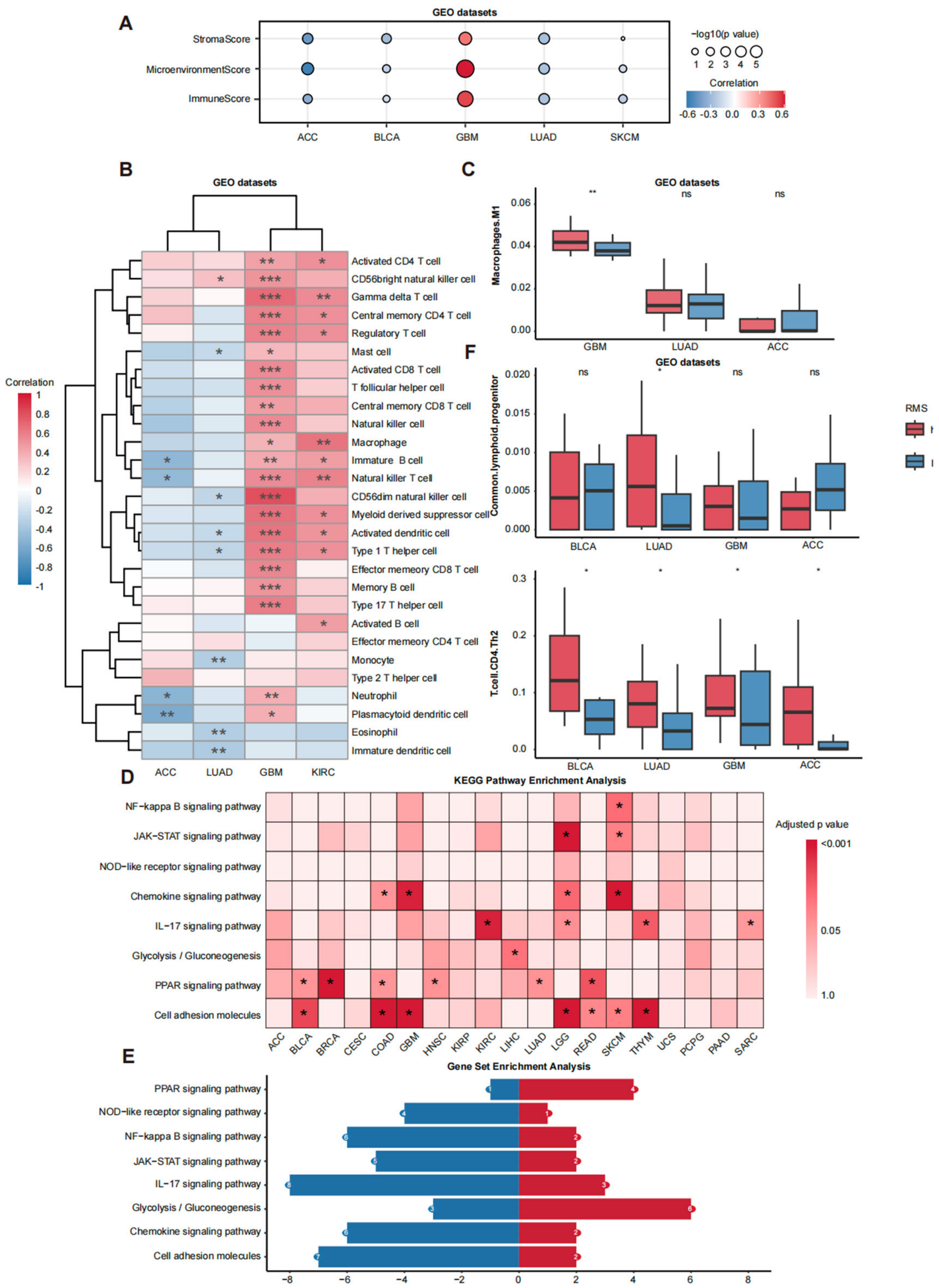

Figure. S8. Comprehensive multi-algorithm analysis of the correlation between RMS and TIME.

(A) Correlation analysis of RMS with ImmuneScore, StromaScore, and MicroenvironmentScore in GEO datasets (quantified by the xCell algorithm) (B) Spearman correlation heatmap between RMS and infiltration levels of 28 immune cell types in GEO datasets (quantified by the ssGSEA

algorithm). Statistical significance was assessed using a two-tailed asymptotic t-test with Holm-Bonferroni correction. *$p < 0.05$, **$p < 0.01$; ***$p < 0.001$ (C) Differences in immune infiltration levels of Macrophages M1 cells in GEO datasets (quantified by the quanTIseq algorithm) between risk RMS groups across 3 cancers (ns: not statistically significant). **$p < 0.01$ (D) KEGG pathway enrichment analysis of M1 macrophage-related pathways among 19 cancer risk RMS groups (E) Gene set enrichment analysis (GSEA) of M1 macrophage-related pathways among 19 cancer risk RMS groups (F) Differences in immune infiltration levels of Common.lymphoid.progenitor (upper) and T.cell.CD4.Th2 cells (lower) in GEO datasets (immune infiltration data are from TIMER website) between risk RMS groups across 4 cancers (ns: not statistically significant). *$p < 0.05$.

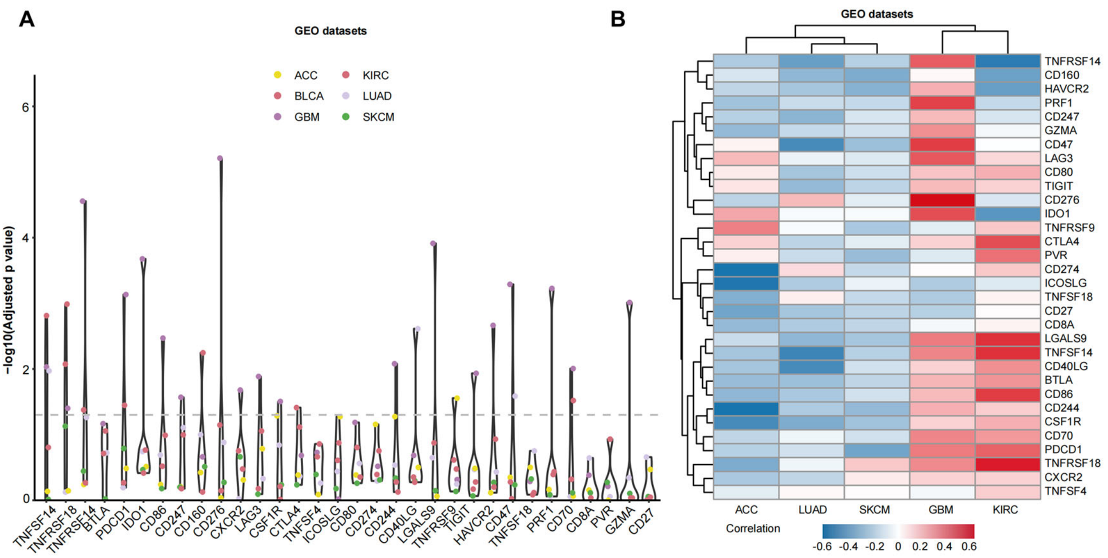

Figure. S9. Potential roles of RMS in tumor immunotherapy in GEO datasets.

(A) Differential analysis of immune checkpoint molecules between risk RMS groups across 6 cancers (Wilcoxon test; dashed line indicates adjusted p value < 0.05) (B) Spearman correlation heatmap between RMS and expression levels of immune checkpoint molecules.

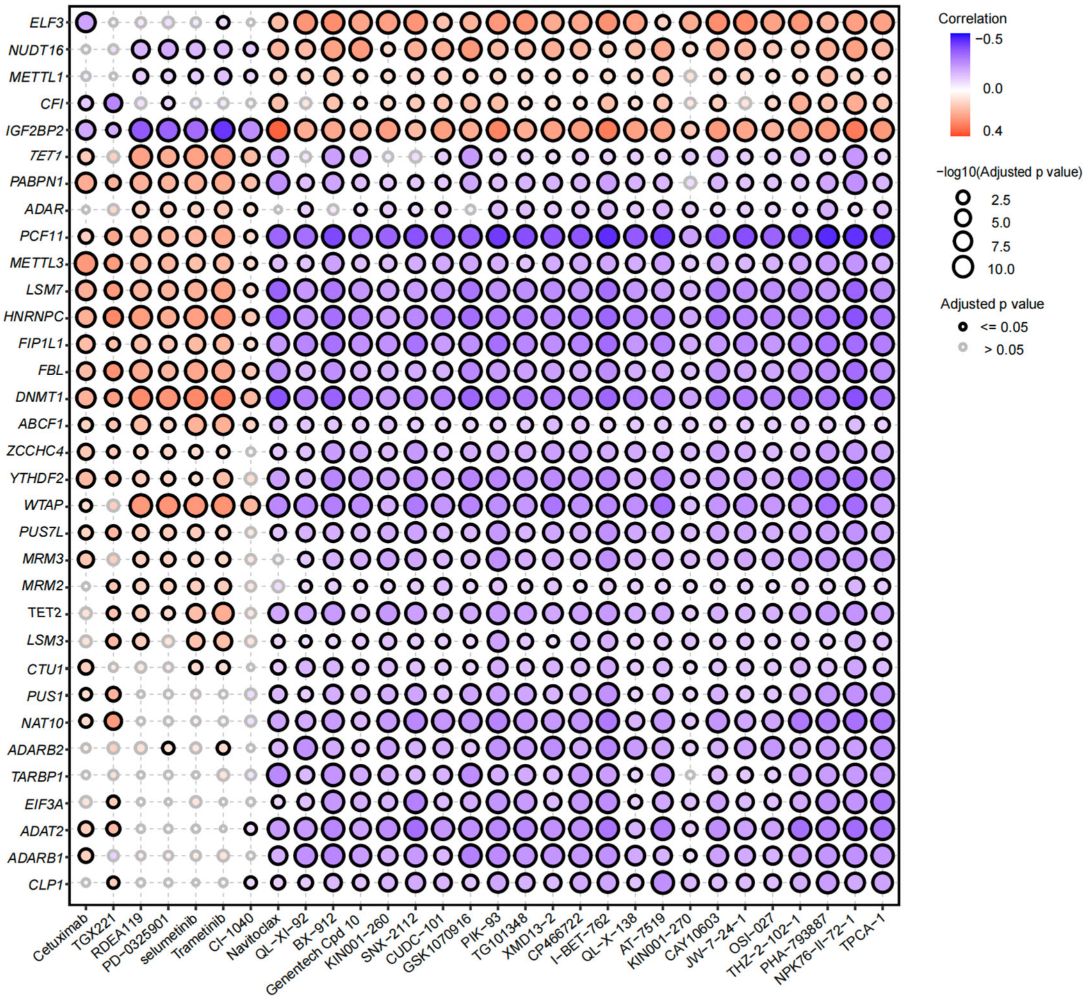

Figure. S10. Sensitivity analysis of RMS-related drug.

GSCALite analysis identifies the top 30 drugs potentially associated with RMS-related RNA modification genes.

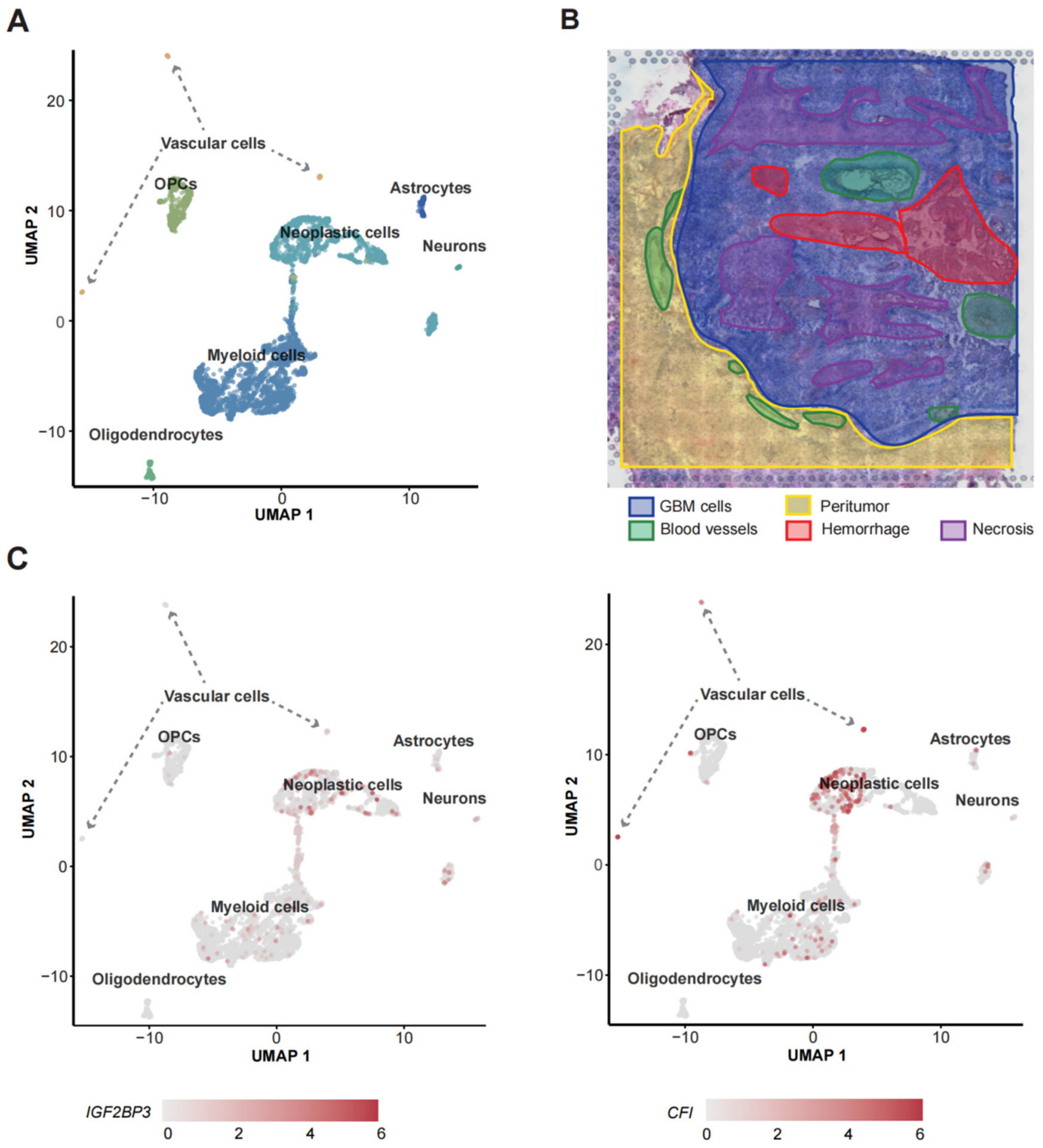

Figure. S11. Single-cell and spatial transcriptomic expression analyses in GBM.

(A) UMAP plot of 7 clustered cell types in GBM (B) Histopathological regions of stained GBM tissue sections (C) UMAP plot of the distribution of IGF2BP3 and CFI across GBM cell types.
